# Brain transcriptomics highlight abundant gene expression and splicing alterations in non-neuronal cells in aFTLD-U

**DOI:** 10.1007/s00401-025-02919-x

**Published:** 2025-08-10

**Authors:** Sara Alidadiani, Júlia Faura, Sarah Wynants, Nele Peeters, Marleen Van den Broeck, Linus De Witte, Rafaela Policarpo, Simon Cheung, Cyril Pottier, Nikhil B. Ghayal, Merel O. Mol, Marka van Blitterswijk, Evan Udine, Mariely DeJesus-Hernandez, Matthew Baker, NiCole A. Finch, Yan W. Asmann, Jeroen G. J. van Rooij, Aivi T. Nguyen, R. Ross Reichard, Alissa L. Nana, Oscar L. Lopez, Adam L. Boxer, Howard J. Rosen, Salvatore Spina, Jochen Herms, Keith A. Josephs, Ronald C. Petersen, Robert A. Rissman, Annie Hiniker, Lee-Cyn Ang, Lea T. Grinberg, Glenda M. Halliday, Bradley F. Boeve, Neill R. Graff-Radford, Harro Seelaar, Manuela Neumann, Julia Kofler, Charles L. White, William W. Seeley, John C. van Swieten, Dennis W. Dickson, Ian R. A. Mackenzie, Wouter De Coster, Rosa Rademakers

**Affiliations:** 1 Department of Biomedical Sciences, University of Antwerp, Building V 0.10, Universiteitsplein 1 2610 Antwerp, Belgium; 2 Applied and Translational Neurogenomics, VIB Center for Molecular Neurology, VIB, Antwerp, Belgium; 3 Department of Pathology, Vancouver Coastal Health, Vancouver, BC Canada; 4 Department of Neuroscience, Mayo Clinic, Jacksonville, FL USA; 5 Department of Neurology, Washington University School of Medicine, St. Louis, MO USA; 6 NeuroGenomics and Informatics Center, Washington University School of Medicine, St. Louis, MO USA; 7 Alzheimer Center, Department of Neurology, Erasmus University Medical Center, Rotterdam, The Netherlands; 8 Department of Quantitative Health Sciences, Mayo Clinic, Jacksonville, FL USA; 9 Department of Laboratory Medicine and Pathology, Mayo Clinic, Rochester, MN USA; 10 Department of Neurology, UCSF Weill Institute for Neurosciences, University of California San Francisco, San Francisco, CA USA; 11 Department of Neurology, University of Pittsburgh, Pittsburg, PA USA; 12 Center for Neuropathology and Prion Research, University Hospital Munich, Ludwig–Maximilians-University, Munich, Germany; 13 German Center for Neurodegenerative Diseases (DZNE), Munich, Germany; 14 Munich Cluster of Systems Neurology (SyNergy), Munich, Germany; 15 Department of Neurology, Mayo Clinic, Rochester, MN USA; 16 Alzheimer’s Therapeutic Research Institute, Keck School of Medicine, University of Southern California, San Diego, CA USA; 17 Department of Pathology, University of California San Diego, La Jolla, CA USA; 18 Department of Pathology, University of Southern California, Los Angeles, CA USA; 19 Department of Pathology and Laboratory Medicine, University of Western Ontario, London, ON Canada; 20 Department of Pathology, London Health Sciences Center, Western University, London, ON Canada; 21 University of Sydney, Sydney, Australia; 22 Department of Neurology, Mayo Clinic, Jacksonville, FL USA; 23 Molecular Neuropathology of Neurodegenerative Diseases, DZNE Tuebingen, Tübingen, Germany; 24 Department of Neuropathology, University Hospital of Tuebingen, Tübingen, Germany; 25 Department of Pathology, University of Pittsburgh, Pittsburg, PA USA; 26 Division of Neuropathology, University of Texas Southwestern Medical Center, Dallas, TX USA; 27 Department of Pathology, University of California, San Francisco, San Francisco, CA USA; 28 Department of Pathology and Laboratory Medicine, University of British Columbia and Vancouver General Hospital, Vancouver, BC Canada

**Keywords:** aFTLD-U, Glial cells, Sonic hedgehog signaling, Mitochondria, Transcriptomics, Splicing

## Abstract

Atypical frontotemporal lobar degeneration with ubiquitin-positive inclusions (aFTLD-U) is a rare cause of frontotemporal lobar degeneration (FTLD), characterized postmortem by neuronal inclusions of the FET family of proteins (FTLD-FET). The recent discovery of TAF15 amyloid filaments in aFTLD-U brains represents a significant step toward improved diagnostic and therapeutic strategies. However, our understanding of the etiology of this FTLD subtype remains limited, which severely hampers translational research efforts. To explore the transcriptomic changes in aFTLD-U, we performed bulk RNA sequencing on the frontal cortex tissue of 21 aFTLD-U patients and 20 control individuals. Cell-type deconvolution revealed loss of excitatory neurons and a higher proportion of astrocytes in aFTLD-U relative to controls. Differential gene expression and co-expression network analysis, adjusted for the shift in cell-type proportions, showed dysregulation of mitochondrial pathways, transcriptional regulators, and upregulation of the Sonic hedgehog (Shh) pathway, including the *GLI1* transcription factor, in aFTLD-U. Overall, oligodendrocyte and astrocyte-enriched genes were significantly over-represented among the differentially expressed genes. Differential splicing analysis confirmed the dysregulation of non-neuronal cell types with significant splicing alterations, particularly in oligodendrocyte-enriched genes, including myelin basic protein (*MBP*), a crucial component of myelin. Immunohistochemistry in frontal cortex brain tissue also showed reduced myelin levels in aFTLD-U patients compared to controls. Together, these findings highlight a central role for glial cells, particularly astrocytes and oligodendrocytes, in the pathogenesis of aFTLD-U, with disruptions in mitochondrial activity, RNA metabolism, Shh signaling, and myelination as possible disease mechanisms. This study offers the first transcriptomic insight into aFTLD-U and presents new avenues for research into FTLD-FET.

**Supplementary Information:**

The online version contains supplementary material available at 10.1007/s00401-025-02919-x.

## Introduction

Frontotemporal lobar degeneration (FTLD) is an umbrella term that groups several neurodegenerative diseases characterized by progressive nerve cell loss, primarily affecting the frontal and temporal lobes [51, 69]. Clinically, these disorders manifest as frontotemporal dementia (FTD) with three distinct presentations: behavioral-variant frontotemporal dementia (bvFTD) [87], semantic variant primary progressive aphasia (svPPA), and nonfluent variant primary progressive aphasia (nfvPPA) [29, 85]. FTD can also present with an extrapyramidal movement disorder, leading to clinical diagnoses of progressive supranuclear palsy (PSP), corticobasal syndrome (CBS) [43], or the combined phenotype of bvFTD and amyotrophic lateral sclerosis (FTLD-ALS) [37, 72].

Beyond its clinical heterogeneity, FTLD is neuropathologically classified based on the accumulation of specific protein inclusions. The three major subtypes are characterized by inclusions of tau (FTLD-tau), TAR DNA-binding protein 43 (TDP-43) (FTLD-TDP), and the FET family of proteins (FTLD-FET), with wide variation in clinical presentation and prognosis [66]. FTLD-FET is the least common subtype, accounting for 5–10% of all FTLD patients; however, its exact frequency remains unknown, as neuropathological subtypes can only be diagnosed at autopsy [75, 92].

While the field has historically referred to the FTLD-FET subtype as FTLD-FUS, it has been known for several years that FTLD-FUS inclusions also display immunoreactivity against transportin 1 (TNPO1), along with several other DNA/RNA-binding proteins that use TNPO1 as their import receptor, including Ewing’s sarcoma protein (EWS) and TATA-binding protein-associated factor 15 (TAF15), which together with FUS make up the FET family of proteins [24, 73, 108]. FET proteins are multifunctional heterogeneous nuclear ribonucleoproteins (hnRNP) that shuttle between the nucleus and cytoplasm, playing essential roles in RNA transcription, splicing, and DNA repair [60, 91]. In addition, cytoplasmic mislocalization and aggregation of other hnRNPs were also reported in FTLD-FET [26]. Cryogenic electron microscopy (cryo-EM) recently solved the structure of the amyloid filaments extracted from the brains of four FTLD-FET patients, which were found to be TAF15, lending additional support to refer to this FTLD pathological subtype as FTLD-FET [65, 108]. The extent to which the pathogenic mechanism underlying FTLD-FET is related to TAF15, FUS, or a combination of dysfunctional hnRNPs is unknown.

Three subtypes have been identified within FTLD-FET: atypical FTLD with ubiquitin inclusions (aFTLD-U), neuronal intermediate filament inclusion body disease (NIFID), and basophilic inclusion body disease (BIBD) [64, 74]. These subtypes differ in the morphology, subcellular localization, and anatomic distribution of FET inclusions, as well as immunoreactivity for other aggregating proteins, such as α-internexin (in the case of NIFID). Among the FTLD-FET subtypes, aFTLD-U is the most common, and it stands out for its characteristic clinical presentation of severe and progressive early-onset bvFTD, often with psychiatric symptoms, without language or motor problems [63, 90, 102]. Pathogenic variants in *FUS* and *TAF15* are known causes of ALS but have not been identified in aFTLD-U patients [11, 16, 52, 88, 111]. In fact, nearly all aFTLD-U patients appear sporadic, suggesting a complex disease etiology.

Despite significant progress in characterizing the neuropathological features of FTLD-FET, a basic understanding of its etiology remains lacking, thereby severely hampering translational research efforts. To gain deeper insight into the molecular underpinnings of FTLD-FET, we performed bulk short-read RNA sequencing of frontal cortex tissue to investigate gene expression and splicing alterations. Notably, our findings indicate that most expression and splicing changes occur not in neurons but in astrocytes and oligodendrocytes. We also observed dysregulation of the Sonic Hedgehog signaling pathway and the *GLI1* transcription factor, which have not been previously implicated in aFTLD-U. This suggests a previously underappreciated role for glial dysfunction in the pathogenesis of aFTLD-U and potentially within the broader FTLD-FET spectrum.

## Materials and methods

### RNA sequencing

RNA was extracted from frontal-cortex tissue using the RNeasy Plus mini kit (Qiagen), and RNA quality and quantity were assessed using an Agilent 2100 Bioanalyzer and the RNA Nano Chip (Agilent Technologies). Only samples with an RNA integrity number (RIN) above seven were included in the study. Samples from 21 aFTLD-U patients and 20 neuropathologically normal controls passed quality control and were designated as the discovery cohort for downstream analysis (Supplementary Table 1). Patients were ascertained from multiple sites, including Erasmus Medical Center (*n* = 6), Ludwig-Maximilians University (*n* = 2), Mayo Clinic Brain Bank (*n* = 5), the University of Sydney (*n* = 3), the University of California San Francisco (*n* = 3), and the University of British Columbia (*n* = 2); all controls were ascertained at the Mayo Clinic Brain Bank (*n* = 20). Due to limited sample availability, the final study cohort was not systematically matched for age and sex (aFTLD-U: 17 male/4 female, mean age at death 49.2 years; controls: 7 male/13 female, mean age at death 82.4 years). Library preparation was performed using Illumina TruSeq mRNA v2 kit and sequenced at 10 samples/lane as paired-end 101 base pair reads on the Illumina HiSeq4000. Read quality was assessed with FastQC, followed by read trimming and filtering with Trimmomatic (with parameters: HEADCROP:10, MINLEN:36) before spliced alignment to the reference genome (GRCh38) using HISAT2 [44]. Gene-level expression was quantified using HTSeq count, in unstranded mode, using the GTF file for GRCh38 from Ensembl [3].

### Cell-type proportion estimation

The CIBERSORTx cell-type deconvolution method was used to estimate the proportions of different brain cell types in bulk RNA-Seq data [77]. The input for CIBERSORTx was read counts converted to Reads Per Kilobase per Million mapped reads (RPKM), normalized with the conditional quantile normalization (CQN) method [78]. We used expression data of 10,319 human adult frontal cortex nuclei (10× snRNA-seq) from the study of Lake et al. for the CIBERSORTx signature matrix to represent the expression profiles of the major brain cell types [53]. The signature matrix contained 9576 genes distinguishing six human brain cell types (astrocytes, endothelial cells, microglia, oligodendrocytes, excitatory and inhibitory neurons) [53, 105]. Since CIBERSORTx is known to have difficulties estimating lowly abundant cell types [80], we did not use the estimated microglial proportion for the downstream analyses. We used the Wilcoxon rank-sum test with Bonferroni adjusted *P *value to test whether the cell type proportions differed significantly between aFTLD-U patients and controls. The distribution of cell-type proportions was visualized using ggplot2 [116].

### Differential expression analyses

Statistical analyses and plots were generated using R packages from CRAN and Bioconductor. Differential expression analysis was performed using DESeq2 [61]. The genes with fewer than 20 samples containing at least 10 supporting reads were excluded, leaving 17,929 genes for analysis. To assess global transcriptional differences between aFTLD-U and control samples, we performed principal component analysis (PCA) using variance-stabilized counts from DESeq2.

To test for differential expression, the first model included RIN, sex, age at death, experimental batch, and disease group as covariates. To account for differences in cell-type compositions in our model, a PCA was performed on the estimated cell-type proportions obtained from CIBERSORTx. The first three principal components (PCs), which together explained over 90% of the variance, were included as covariates in a second model, in addition to the covariates mentioned above. Shrinkage was applied to the fold changes using the *apeglm* method for effect size shrinkage [119], and adjusted *P* values were determined based on multiple testing corrections with the Benjamini–Hochberg procedure. Genes were considered significantly differentially expressed genes (DEGs) when their adjusted *P* value was below 0.05. We considered the genes with log2FC > 0.3 upregulated and those with log2FC < − 0.3 as downregulated. DEGs were subsequently used in a pathway analysis separately for up- and downregulated genes with the enrichR package [50], using “GO Biological Process 2023” gene sets to determine pathway enrichment [2]. All expressed genes were used as the background set. DEGs were also used as input for a STRING protein–protein interaction (PPI) network analysis [107]. We used text-mining, experiments, databases, and co-expression as interaction sources to construct a full STRING network. Interacting proteins with a high confidence of 0.7 were used and unconnected nodes and nodes with only two connections were excluded. The visualizations were generated using the ggplot2 and plotEnrich packages.

### Weighted gene co-expression network analysis

We used the R package weighted gene co-expression network analysis (WGCNA) to identify sets of highly correlated genes (modules) [54], using residual expression values as input. These residuals were obtained after CQN normalization [32], followed by adjustment for covariates through a multivariable linear regression model. Only cell type composition (using the first three PCs of the proportions of cell types), RIN, and experimental batch could be used as covariates; age and gender were too unbalanced between the groups and could not be included in the generation of residual values for this specific analysis. We selected a power of 14 to achieve a scale-free topology. We used the blockwiseModules function with a minimum module size of 30 genes, a merge height of 0.35, a Pearson correlation coefficient, and the signed hybrid network type. Modules generated using these settings were represented by their first PC (module eigengene) and a unique color. Genes that did not fulfill the criteria for any of the modules were assigned to the gray module. To assess the correlation of modules to aFTLD-U, we defined the controls as 1 and the aFTLD-U group as 2. Highly correlated modules associated with aFTLD-U were annotated using the enrichR package [50], using “GO Biological Process 2023” gene sets to determine pathway enrichment.

### Differential splicing analysis using LeafCutter

LeafCutter (v0.2.6) was used to identify differentially spliced genes between conditions [56]. It is optimized for finding novel splicing by clustering overlapping splice junction reads and comparing the contribution of each junction between conditions. Importantly, LeafCutter does not estimate isoform abundance or exon inclusion levels but instead captures changes in local splicing events through the construction of intron clusters, wherein overlapping introns are connected by the splice junction(s) they share. In this analysis, splice junctions from the STAR [21] aligner were extracted using Regtools [15]. STAR was selected over HISAT2 due to its enhanced sensitivity in detecting novel splice junctions [5, 21]. Junctions were extracted with the following parameters: minimum anchor length of 8 bp, minimum intron length of 50 bp, and maximum intron length of 500,000 bp. To identify differential splicing events, a Dirichlet-Multinomial generalized linear model was applied using two separate models: one adjusting for the covariates of age, sex, RIN, and experimental batch, and the second model additionally included the first three PCs of the proportions of cell types.

Originally, cell-type proportions were estimated using CIBERSORTx on HISAT2-aligned read counts. However, to ensure consistency with the STAR-aligned reads used for splicing analysis, we re-ran CIBERSORTx on STAR-aligned read counts. To assess the consistency between aligners, we computed pairwise correlations between cell-type proportion estimates derived from HISAT2- and STAR-aligned reads. The normality of the data was assessed using the D’Agostino–Pearson omnibus test, and accordingly, either Pearson or Spearman correlation coefficients were computed (Supplementary Fig. 1a–e). PCs were then recalculated from the updated STAR-based estimates, and the first three PCs, which explained over 90% of the variance in cell-type proportions, were included as covariates in the extended splicing model. The annotation of the intron junctions was performed using the GENCODE v42 reference transcriptome and subsequently classified into one of the following categories: annotated, novel annotated pair, novel acceptor (cryptic 3’), novel donor (cryptic 5’), novel acceptor and novel donor (cryptic_unanchored), ambiguous gene and unannotated (“unknown_strand”). In downstream analyses, junctions that mapped to more than one gene (“ambiguous gene”) and unannotated events were not considered.

The intron clusters were defined to be differentially spliced if they had an FDR < 0.05 and at least one intron excision event with a percentage spliced in difference |ΔPSI|≥ 5%. Significant clusters were classified as cassette exons, using leafviz, based on whether the cluster contained three splice junctions in the correct orientation, with two junctions flanking a central exon (inclusion junctions) and a third junction spanning the length of the cluster (skipping junction), and whether the inclusion and/or skipping junctions were annotated in GENCODE (v42).

Pathway and STRING PPI network analyses were conducted using the same methodology as those used for the differential expression analyses.

### Assignment of genes to brain cell types based on expression specificity

To assess whether differentially expressed or spliced genes were enriched in expression in specific brain cell types, we performed a cell type enrichment analysis using publicly available data from the Human Protein Atlas (https://www.proteinatlas.org/) [42, 99]. For each analysis, two gene sets were defined: (1) a background list comprising all genes detected in the respective analysis and (2) a list of statistically significant genes (e.g., differentially expressed or spliced).

Normalized expression values (nTPM) were used to quantify gene expression across annotated brain cell types. A gene was classified as cell type-enriched if it exhibited a *z*-score ≥ 2 in a single cell type, and a log2FC > 0.3 between the top two most highly expressing cell types for that gene. This approach identified genes showing strong relative expression enrichment in a particular cell type.

Using a hypergeometric test, we determined whether there was an enrichment of genes assigned to a specific cell type among the significantly expressed or spliced genes. For each cell type, the number of enriched genes in the significant list was compared against the number of enriched genes in the background list. *P* values were computed using the hypergeometric distribution, and multiple testing corrections were performed using the Bonferroni method.

Additionally, to visualize cell-type expression patterns, we generated heatmaps displaying the expression of the top 50 differentially expressed and spliced genes across six major brain cell types: excitatory neurons, inhibitory neurons, astrocytes, microglia, oligodendrocytes, and oligodendrocyte precursor cells (OPCs). Heatmaps were generated using the R package *pheatmap* [48].

### Quantitative PCR

Two independent cohorts of postmortem frontal cortex tissue from aFTLD-U patients and controls were included (Supplementary Table 1). The validation cohort consisted of 11 aFTLD-U patients and 8 neuropathologically normal control individuals, already included in the bulk RNA sequencing data. The replication cohort comprised 11 additional aFTLD-U patients and 10 controls from different sites, including Erasmus Medical Center (*n* = 3 aFTLD-U, *n* = 4 controls), Mayo Clinic Brain Bank (*n* = 1 aFTLD-U, *n* = 1 control), London Health Sciences Center (*n* = 1 aFTLD-U), University of California San Francisco (*n* = 1 aFTLD-U), University of California San Diego (*n* = 1 aFTLD-U), University of Pittsburgh (*n* = 1 aFTLD-U), University of Texas Southwestern (*n* = 2 aFTLD-U, *n* = 1 controls), and the University of British Columbia (*n* = 1 aFTLD-U, *n* = 4 controls). Similar to the discovery cohort, aFTLD-U and controls were not matched for age at death and sex (Supplementary Table 1).

RNA was extracted from the selected samples using the RNeasy Mini kit (Qiagen). Quality control was performed using a fragment analyzer (Agilent), and the concentration was measured with a Qubit (ThermoFisher Scientific). Samples above RIN 5 were included for qPCR. 500 ng of RNA were reverse transcribed using the iScript™ complementary DNA (cDNA) (Bio-Rad) synthesis kit. Real-time quantitative PCRs (qPCRs) were performed using the *Power* SYBR™ Green PCR Master Mix (Applied Biosystems) on a QuantStudioTM 6 Flex Real-time PCR system (Applied Biosystems). The primer sequences can be found in Supplementary Table 2.

The gene expression levels were quantified using the comparative Ct (2^–ΔΔCt^) method. The expression of the target genes was normalized to the geometric mean of two housekeeping genes, RPLP0 and UBE2DE, and the results are presented as relative quantity (RQ) values compared to those of the control group. For genes associated with alternative splicing events, two sets of event-specific primers were designed for each gene to distinguish between alternative events of interest. To enable a direct comparison between qPCR-based splicing estimates and PSI values from Leafcutter, Percentage Spliced-In (PSI) was calculated for each sample. PSI was determined by dividing the RQ value of one event by the sum of the RQ values of both events of a gene. This approach enabled a quantitative assessment of event abundance and validated the splicing changes observed in transcriptomic analyses.

In all experiments, data are represented as mean ± SD. The normality of the data was assessed using the D’Agostino–Pearson omnibus test. For comparisons between the two groups, as appropriate, either Student’s *t* test (for normally distributed data) or the Mann–Whitney *U* test (for non-normally distributed data) was used. Correlation analyses were performed using either Pearson’s correlation coefficient (for normally distributed data) or Spearman’s correlation coefficient (for non-normally distributed data), based on the normality test results. Statistical analyses were conducted using GraphPad Prism (GraphPad Software, San Diego, CA). A *P* value < 0.05 was considered statistically significant.

### MBP immunohistochemistry

Immunohistochemistry was performed on four micrometer thick sections of formalin fixed, paraffin embedded postmortem brain tissue from deep frontal lobe white matter (level of the genu of the corpus callosum) from 9 individuals with aFTLD-U, 7 neuropathologically normal controls, and 9 FTLD-TDP patients obtained from the University of British Columbia brain bank (Supplementary Table 1). Immunostaining was performed using the DAKO automated immunostainer, with a primary antibody that recognizes myelin basic protein (MBP) (Sigma Aldrich, anti-MBP, 1:200). Slides were digitally scanned at 40× magnification and the amount of specific staining within a representative 7 mm^2^ region (3 mm diameter circle) in the center of the section was quantified using the Leica Aperio ImageScope Positive Pixel Count algorithm. The percentage of stained surface area was determined by calculating the proportion of total pixels within the area of interest with at least medium positivity. Statistical analysis was performed using Kruskal–Wallis test, followed by Dunn’s multiple comparisons with Bonferroni correction. Data are reported as median, with 95% confidence intervals.

### Luxol fast blue myelin staining

Standard Luxol fast blue stain with hematoxylin and eosin counter stain (LFB/HE) was performed on six micrometer thick sections of formalin-fixed, paraffin-embedded material to visualize overall myelin distribution [62]. The stained sections were from the same region as used for the MBP immunohistochemistry and performed on selected individuals, chosen to have MBP staining levels closest to the group mean.

## Results

### Altered cell-type composition and dysregulated gene expression in mitochondrial and Sonic hedgehog (Shh) pathways in aFTLD-U

**Fig. 1:**
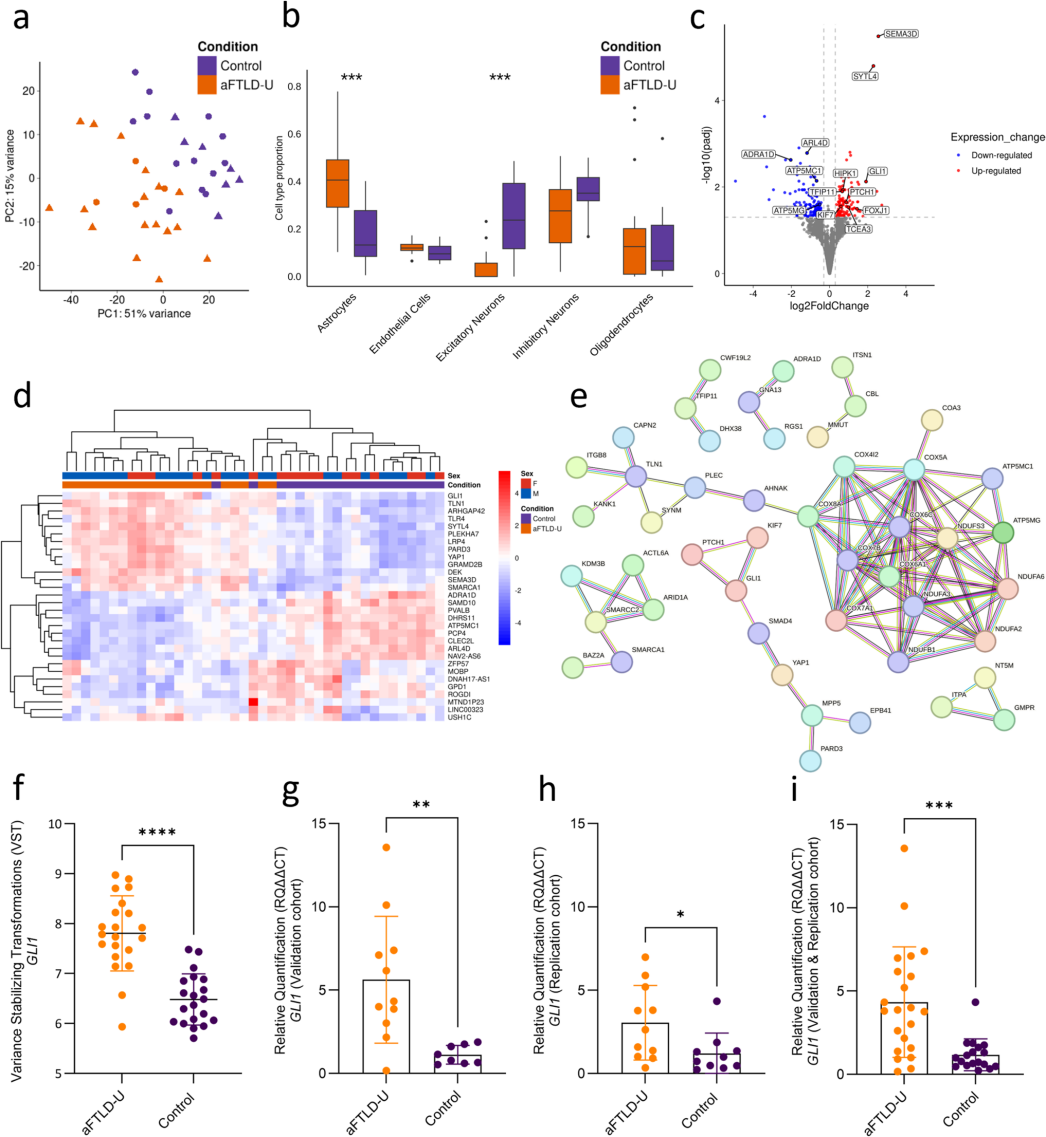
Transcriptomic analyses in aFTLD-U versus controls. (**a**) Principal component (PC) analyses of frontal cortex bulk transcriptome data in 21 aFTLD-U patients (orange) and 20 control individuals (purple). Females are indicated with a circle, and males with a triangle. (**b**) Cell-type deconvolution shows estimated proportions of five cell types by CIBERSORTx (astrocytes, endothelial cells, excitatory neurons, inhibitory neurons, and oligodendrocytes). Wilcoxon rank-sum test, Bonferroni adjustment-*p* value (***P ≤ 0.001) (**c**) Volcano plot representing the differentially expressed genes in aFTLD-U patients versus controls, with adjustment for cell-type proportions. The fold change is presented in a log2 scale at the *x*-axis, while the adjusted *P* value is presented on the *y*-axis on a − log10 scale. (**d**) Heat map of the top 30 most significant genes from the differential gene expression analyses with adjustment for cell-type proportions. (**e**) STRING protein–protein interaction (PPI) network analysis on the DEGs. Disconnected nodes and nodes with only two connections are removed. We used a full STRING network; the edges represent both functional and physical protein associations. Line color indicates the type of interaction evidence. All colored nodes represent query proteins and the first shell of interactors. (**f**) Variance-stabilized transformed (VST) expression values of *GLI1* from bulk RNA sequencing data showed significantly higher expression in aFTLD-U patients compared to controls (*t* test, *p* value < 0.0001). (**g**) Relative quantification (RQ) values from qPCR validation for *GLI1* showed an increased level of *GLI1* in aFTLD-U patients compared to the controls (*t* test, *p* value = 0.0042). (**h**) Relative quantification (RQ) values from qPCR replication of *GLI1* expression, confirming increased expression in aFTLD-U patients (Mann–Whitney *U* test, *p* value = 0.0295). (**i**) A combined analysis of the validation and replication cohorts for *GLI1* expression confirmed increased expression in aFTLD-U patients (Mann–Whitney *U* test, *p* value = 0.0003). In all plots, each dot represents an individual sample, and the data are represented as mean ± SD

We performed RNA sequencing on the frontal cortex tissue of 21 aFTLD-U patients and 20 neuropathologically normal controls (Supplementary Table 1). The selection of patients was based on tissue availability at the time of the experiment and RIN, with representation of patients from multiple sites and only including aFTLD-U patients with Caucasian ethnicity and with the classical clinical presentation of bvFTD. PCA revealed global transcriptional differences between aFTLD-U patients and controls (Fig. 1a). Since an altered cellular proportion resulting from neurodegeneration was expected, we first used CIBERSORTx to perform cell-type deconvolution for major cell types in the brain. Relative to controls, we found a significant loss of excitatory neurons (mean ± SD: 0.03 ± 0.06 in aFTLD-U vs. 0.24 ± 0.03 in controls, Wilcoxon rank-sum test, Bonferroni adjustment-*p* value = 0.0002) as well as a larger proportion of astrocytes in aFTLD-U patients as compared to controls (mean ± SD: 0.41 ± 0.18 in aFTLD-U vs. 0.17 ± 0.12 in controls, Wilcoxon rank-sum test, Bonferroni adjustment-*p* value = 0.00008) (Fig. 1b).

Without adjustment for cell type proportions, 3998 genes were differentially expressed between aFTLD-U patients and controls (adjusted *p* value < 0.05), including 2096 genes upregulated (log2FC > 0.3) and 1902 genes downregulated (log2FC < − 0.3) (Supplementary Fig. 2 and Supplementary Table 3). However, when adjusting for cell-type proportions by using the first three PCs of the cell-type proportions estimated by CIBERSORTx, 271 genes were significantly different (adjusted *p* value < 0.05), including 134 genes upregulated (log2FC > 0.3), and 137 genes downregulated (log2FC < − 0.3) (Fig. 1c and Supplementary Table 4). *SYTL4* and *SEMA3D* (log_2_FC = 2.29, adjusted *p* value = 1.60E−05 and log_2_FC = 2.55, adjusted *p* value = 3.30E-06, respectively) were the most significantly upregulated genes in aFTLD-U. To further investigate the expression changes, we generated a heatmap for the top 30 DEGs based on the adjusted *P* value. The heatmap showed a clear separation between aFTLD-U and control samples, supporting the presence of condition-specific transcriptional signatures (Fig. 1d).

Pathway enrichment analyses were performed on DEGs identified after correction for cell type proportions. Upregulated genes were enriched for terms related to transcription regulation including DNA-templated transcription (adjusted *p* value = 8.12E−05), regulation of transcription by RNA Polymerase II (adjusted *p* value = 1.63E−04), and signaling pathways including regulation of Smoothened signaling pathway (adjusted *p* value = 5.52E−03), while downregulated genes showed enrichment for terms related to mitochondrial electron transport including aerobic electron transport chain (adjusted *p* value = 2.16E−13), and mitochondrial respiratory chain (adjusted *p* value = 9.88E−08) (Supplementary Table 6, Supplementary Fig. 3a, b, respectively). The results of pathway enrichment analyses without cell type correction are included in the supplementary results (Supplementary Table 5, Supplementary Fig. 3c, d, respectively). As an alternative approach, we also performed module-level analyses using WGCNA, which identified similar dysregulated pathways. The top negatively correlated module with aFTLD-U was enriched for terms focusing on cellular respiration and proton motive force-driven ATP synthesis (green, Pearson Cor = − 0.43, *p* value = 0.006). A second module, negatively correlated with aFTLD-U, was enriched for terms related to synaptic signaling (blue, Pearson Cor = − 0.41, *p* value = 0.008). The highest positively correlated module was enriched for terms related to the regulation of gene transcription and translation (red, Pearson Cor = 0.41, *p* value = 0.008) (Supplementary Table 7, Supplementary Fig. 4a–c, respectively).

To better visualize the overrepresented pathways and identify protein networks dysregulated in aFTLD-U, we generated STRING PPI networks using the 271 dysregulated genes. This analysis showed a large cluster of proteins involved in mitochondrial function (e.g., COX8A, COX7B, COX6C, NDUFA6, ATP5MG) weakly linked to a group of proteins related to cytoskeletal organization, cell adhesion, and migration (e.g., KANK1, TLN1, AHNAK, SYNM). The second largest protein network was related to various signaling pathway regulators (e.g., KIF7, GLI1, PTCH1, SMAD4, and YAP1) (Fig. 1e). Interestingly, this network included KIF7, GLI1, and PTCH1, which are all crucial elements of the Shh signaling pathway in line with the enrichment term associated with the regulation of smoothened signaling pathway identified above (*smoothened* is a transmembrane protein that is a key component of the Shh pathway). In fact, the transcription factor *GLI1* was among the most upregulated genes in all our analyses (log_2_FC = 2.74, adjusted *p* value = 1.09E−07 without correction for cell type proportions; and log_2_FC = 1.90, adjusted *p* value = 0.007 with correction for cell type proportions) (Fig. 1f).

To validate these findings, we performed qPCR in a subset of aFTLD-U patients and controls included in the RNA-seq study. This analysis confirmed the significant increase of *GLI1* in aFTLD-U patients (*p* value = 0.0042) (Fig. 1g), with a strong correlation of expression values between both techniques (Spearman Cor = 0.81, *p* value = 0.00003) (Supplementary Fig. 5). Next, we evaluated *GLI1* expression in an independent cohort that included an additional 11 aFTLD-U and 10 controls, replicating the significant increase in *GLI1* in aFTLD-U patients (*p* value = 0.02) (Fig. 1h). Analysis of the combined cohort further supported a robust upregulation of *GLI1* in aFTLD-U (*p* value = 0.0003) (Fig. 1i).

Finally, we performed a cell type enrichment analysis to determine whether DEGs were disproportionately associated with specific brain cell types based on their cell type–specific expression pattern. Among the 32 DEGs with cell type–enriched expression, genes associated with both oligodendrocytes and astrocytes were enriched compared to their proportions in the background set of 1356 cell–type–enriched genes. Astrocyte-enriched genes showed a 2.17-fold enrichment among DEGs (a total of 11/32 from DEGs vs. 214/1356 from the background; Bonferroni-adjusted *p* value = 0.04). Also, oligodendrocyte-enriched genes exhibited a significant 1.96-fold enrichment (a total of 14/32 from DEGs vs. 302/1356 from the background; Bonferroni-adjusted *p* value = 0.03) (Supplementary Table 8). Visualization of the top 50 DEGs, using the nTPM values from the major brain cell types in Human Protein Atlas data, revealed many genes with high expression in astrocytes and oligodendrocytes, including 4 astrocyte- and 5 oligodendrocyte-enriched genes (Supplementary Fig. 6, Supplementary Table 8).

### Dysregulation of gene splicing in oligodendrocyte-enriched genes in aFTLD-U

In a differential splicing analysis using LeafCutter, we identified 3509 differentially spliced events distributed across 1432 clusters (FDR < 0.05, |ΔPSI|≥ 5%) corresponding to 1230 unique genes without adjusting for cell type proportions (Supplementary Table 9, Supplementary Figs. 7 and 8a). Of these clusters, 399 were categorized as cassette exons with primary skipped (74.93%), 19.54% were included, and 5.26% were categorized as complex. We also identified 306 novel splicing events, including novel annotated pair (*n* = 123), cryptic 5’ (*n* = 88), cryptic 3’ (*n* = 85), and cryptic unanchored (*n* = 10) (Supplementary Table 9, Supplementary Fig. 8b).

Pathway enrichment analysis on these gene sets revealed associations with neuronal development process, regulation of GTPase activity, and regulation of RNA splicing (Supplementary Table 10, Supplementary Fig. 8c).

**Fig. 2:**
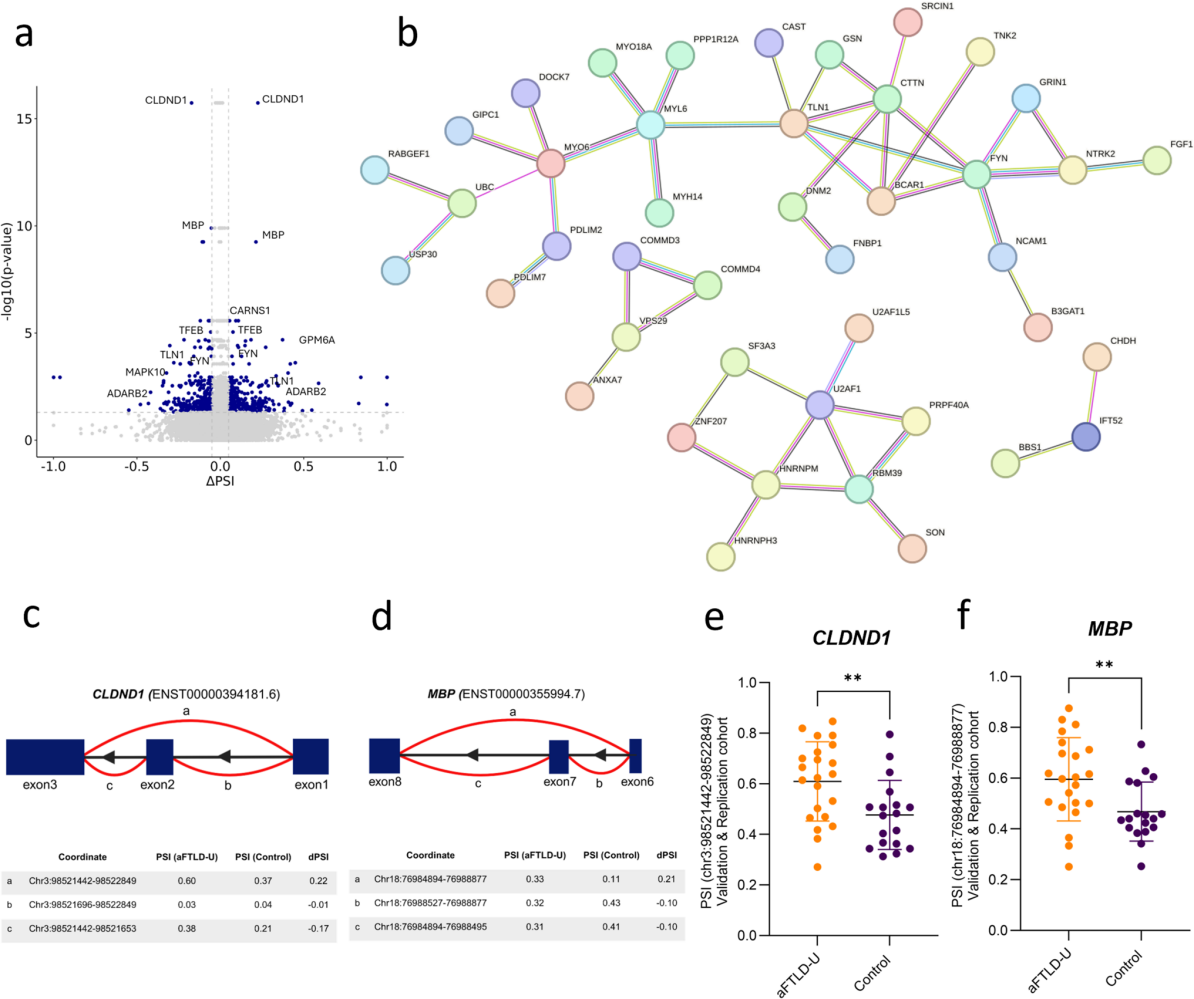
Splicing alteration in aFTLD-U versus controls. (**a**) Volcano plot of differentially spliced events in aFTLD-U versus controls adjusted by cell type proportions. In dark blue, events within a significant cluster (FDR < 0.05) and a |ΔPSI|≥ 5%. (**b**) STRING protein–protein interaction (PPI) network analysis on the significantly differentially spliced genes. Disconnected nodes and nodes with only two connections are removed. (**c**, **d**) Schematic representation of the splicing events observed in *CLDND1* and *MBP*. Exons are represented as dark blue boxes, and splice junctions are shown as curved lines. Figure created with Biorender. Below each schematic, tables display the chromosomal coordinates of the splice junctions, and PSI values for aFTLD-U patients and controls are indicated in the table. (**e**) Combined data of the validation and replication cohorts for *CLDND1* showed significant differences in splicing (chr3:98,521,442–98,522,849) between aFTLD-U patients and controls (*t* test, *p* value = 0.0077) (**f**) Combined data of the validation and replication cohorts for *MBP* showed significant differences in splicing (chr18:76,984,894–76,988,877) between aFTLD-U patients and controls (*t* test, *p* value = 0.0087). In all plots, each dot represents an individual sample, and the data are represented as mean ± SD

To more accurately capture disease-specific splicing changes, we adjusted our model for the first three PCs of the cell-type proportions. After this adjustment, we observed a notable reduction in the number of significant splicing events (Fig. 2a, Supplementary Fig. 8a); however, we still identified 624 differentially spliced events distributed across 249 clusters and 227 unique genes. Among these 249 clusters exhibiting differentially spliced events, 65 were labeled cassette exons. 72.30% of these cassette exons were skipped, 13.84% were included, and 13.84% were complex. Of all the 624 differentially spliced events, 85 were categorized as novel, with novel annotated pair (*n* = 27), cryptic 5’ (*n* = 31), cryptic 3’ (*n* = 20), and cryptic unanchored (*n* = 7) (Supplementary Table 11, Supplementary Fig. 8b). No significant enriched pathways were found for this analysis (Supplementary Fig. 8d, Supplementary Table 12).

To better identify protein networks dysregulated in aFTLD-U, we next generated STRING protein–protein interaction (PPI) networks using the 227 differentially spliced genes (Fig. 2b). This analysis showed a large cluster of proteins involved in cytoskeletal structure, with FYN interacting with other proteins, including NCAM1, NTRRK2, and GRIN1. Additionally, this cluster was linked to the proteins involved in cytoskeletal organization and cell adhesion, including BCAR1, CTTN, and TLN1, which were connected to the myosin family of proteins, with MYO6 as a node. The splicing event in *FYN* corresponds to intron 9 of ENST00000368682.8 and intron 8 of ENST00000368678.8 (chr6:111,696,456–111,699,515, FDR = 0.002, ΔPSI = 0.11). The second cluster from the PPI network was related to proteins that function in splicing, including U2AF1 as a central node, interacting with multiple RNA-binding proteins, including RBM39, PRPF40A, HNRNPM, ZNF207, SF3A3, and SON.

Splicing events in *CLDND1* and *MBP* were the most significant, even after adjustment for cell type proportions (Fig. 2a). Specifically, in the aFTLD-U patients, we observed an increased inclusion of an annotated junction chr3:98,521,442–98,522,849 of *CLDND1* (FDR = 1.84E−16, ΔPSI = 0.30) (Fig. 2c, Supplementary Fig. 9a,b), that results from the skipping of exon 2 in ENST00000507874.5, ENST00000394180.6, ENST00000394185.6, ENST00000394181.6, ENST00000510545.5. In the case of *MBP*, we identified significantly more skipping of exon 7 of the canonical transcript of *MBP* (chr18:76,984,894–76,988,877, FDR = 5.64E−10, ΔPSI = 0.20) (Fig. 2d, Supplementary Fig. 10a, b). We performed qPCR and calculated the PSI values to validate and replicate these findings. In the validation cohort, we confirmed these splicing events in *CLDND1* (*p* value = 0.001) (Supplementary Fig. 9c) and *MBP* (*p* value = 0.003) (Supplementary Fig. 10c) with a strong correlation between the qPCR and RNA-seq values for *CLDND1* (Pearson Cor = 0.88, *p* value = 9.4e−07) (Supplementary Fig. 9e) and *MBP* (Pearson Cor = 0.87, *p* value = 1.7e−06) (Supplementary Fig. 10e). While these splice changes did not reach significance in the replication cohort (*CLDND1*, *p* value = 0.45; *MBP*, *p* value = 0.37) (Supplementary Fig. 9d and 10d, respectively), statistically significant differential splicing was observed in the combined cohort for both genes (*CLDND1, p* value = 0.007; *MBP, p* value = 0.008) (Fig. 2e and f, respectively).

To further explore the cellular specificity of the differentially spliced genes, we performed a cell-type enrichment analysis. We identified 23 cell type–enriched genes among the significant splicing events and 534 cell–type–enriched genes within the background gene set. Notably, 15 from the 23 significantly differentially spliced genes were enriched in oligodendrocytes, which represents a 1.65-fold enrichment, almost reaching statistical significance after multiple testing correction (Bonferroni-adjusted *p* value = 0.06) (Supplementary Table 13). Supporting this, visualization of the top 50 differentially spliced genes using the nTPM values from the Human Protein Atlas data showed that many of these genes (24 out of the 50) are highly expressed in oligodendrocytes with 4 oligodendrocyte-enriched genes (Supplementary Fig. 11, Supplementary Table 13).

### Significant reduction in MBP staining in aFTLD-U

**Fig. 3:**
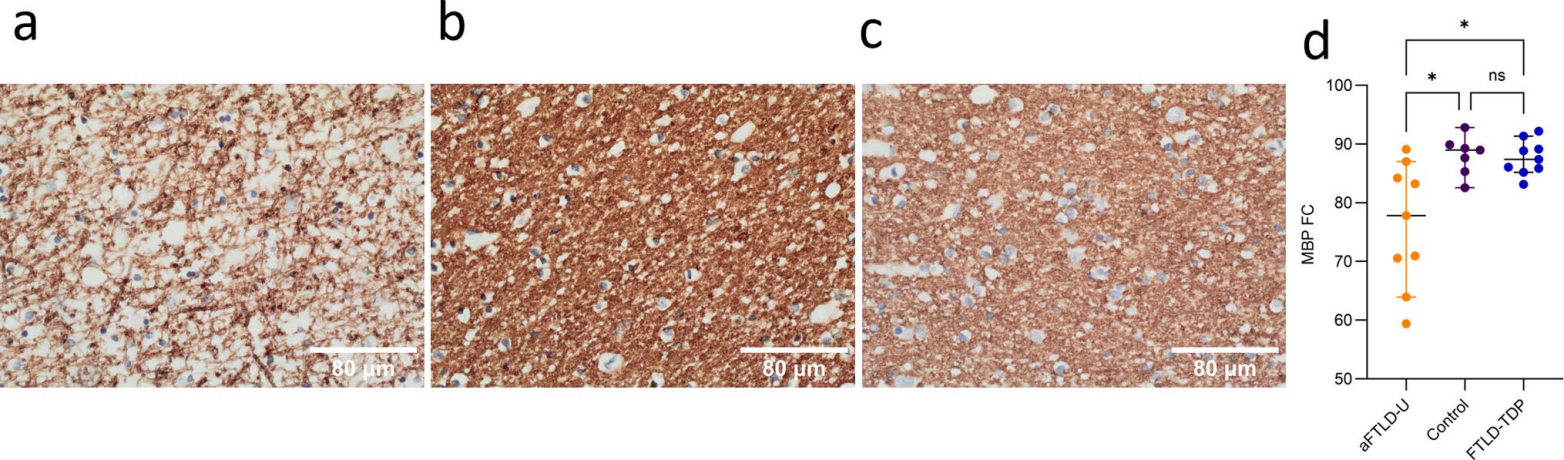
MBP immunohistochemistry. Immunohistochemistry for deep frontal lobe myelin in (**a**) patient with aFTLD-U pathology, (**b**) control, and (**c**) patient with FTLD-TDP pathology, with values closest to the mean for their respective groups. (**d**) Comparison of the percentage surface area of white matter stained for MBP (MBP FC) showing significant reduction in aFTLD-U patients compared to controls (Dunn, Bonferroni-adjusted *p* value = 0.02) and FTLD-TDP patients (Dunn, Bonferroni-adjusted *p* value = 0.04) groups. The greater variability among aFTLD-U patients suggests heterogeneity in myelin loss severity. (**a**–**c**) MBP immunohistochemistry; scale bar, 80 μm. (**d**) Each dot represents one individual. Data is shown as median with 95% confidence interval

As the most differentially spliced genes were key oligodendrocyte genes, including *MBP*, and most differentially spliced genes were enriched in expression in oligodendrocytes, we performed immunohistochemistry to assess myelin content in deep cerebral white matter. Immunohistochemistry for MBP showed a significant reduction in staining in the aFTLD-U group compared to controls and FTLD-TDP patients (Fig. 3a–c, respectively). The mean proportion of surface area stained for MBP in aFTLD-U patients was 76.24 ± 10.56%, while controls and FTLD-TDP had significantly higher mean values of 88.07 ± 3.32% (*p* value = 0.02) for controls and 87.68 ± 2.96% for FTLD-TDP patients (*p* value = 0.04) **(**Fig. 3d**)**. LFB/HE staining in representative individuals from each group confirmed the marked reduction in myelin in an aFTLD-U patient compared to a control and FTLD-TDP patient (Supplementary Fig. 12).

## Discussion

Since the initial description of aFTLD-U as a distinct pathological subtype of FTLD characterized by FUS as the pathological protein almost 15 years ago [75], most progress has been made in the characterization of the pathological aggregates and the comparison with pathology in ALS-FUS, including the change in designation from FTLD-FUS to FTLD-FET and the hypothesis that other mechanisms (potentially upstream of the FET proteins) may induce the protein aggregation in these patients [24, 65, 73]. In this study, we provide insights into the pathophysiological mechanisms underlying aFTLD-U by performing the first unbiased transcriptome sequencing study in this disease, comparing gene expression and alternative splicing patterns in an affected region, the frontal cortex, of aFTLD-U patients and neuropathologically normal individuals.

As expected from the analysis of affected brain tissue, we found robust shifts in brain cell-type abundance using cell-type deconvolution in aFTLD-U, with a notable selective loss of excitatory neurons and a strong and significant increase in the relative abundance of astrocytic markers compared to controls. A similar selective vulnerability of excitatory neurons has been identified in other neurodegenerative diseases. The estimated higher proportion of astrocytes in aFTLD-U patients may be related to astrocyte activation. Astrocytes contribute to the regulation of neuronal activity and are involved in clearing debris and maintaining brain homeostasis. In response to neurodegeneration, they produce pro-inflammatory cytokines, chemokines, and reactive oxygen species that contribute to the state of neuroinflammation [27, 58, 86]. While this may initially be beneficial for removing protein aggregates and dying neurons, sustained inflammatory responses can damage neurons and synapses, ultimately exacerbating neurodegeneration.

Using a differential gene expression approach with correction for age, sex, and cell type distributions, 271 DEGs were identified. The two genes with the most significant difference between aFTLD-U and controls were *SEMA3D,* involved in axon guidance and synapse formation through cytoskeleton reorganization [12, 22, 84] and *SYTL4*, which regulates synaptic vesicle function and intracellular signaling via interaction with Rab GTPases [76, 106].

Focusing on pathway-enrichment analyses of the DEGs and co-expression network analyses, we consistently identified three distinct dysregulated pathways. In aFTLD-U patients, we identified a decrease in the expression of mitochondrial function-related pathways (as compared to controls) and an increase in pathways related to nucleic acid transcription and translation and the regulation of the smoothened signaling pathway.

The extensive mitochondrial dysfunction observed in the aFTLD-U patients included dysregulated genes from the NDUFA and COX gene families, which are essential parts of Complex I and IV and required for the proper functioning of the mitochondrial respiratory chain [81, 98]. Genes encoding for the Mitochondrial ATP synthase (complex V) were also downregulated. There is increasing evidence for mitochondrial dysfunction in a range of neurological diseases, including FTLD-TDP, Alzheimer’s disease, Parkinson’s disease, Huntington’s disease, anxiety, and depression [4, 7, 10, 14, 18, 59, 97, 100, 113, 114]. Mislocalization of FUS to the cytoplasm, as observed in aFTLD-U, was reported to damage mitochondria by inducing mitochondrial fragmentation, and electron microscopy indeed confirmed mitochondrial deficits in FTLD-FET brains [19, 20, 71, 109]. Moreover, a recent study suggests an essential physiological role for FUS in mitochondrial DNA repair through its interaction and recruitment of mitochondrial Ligase IIIα to DNA damage sites within mitochondria, a process which may be compromised due to the loss of normal FUS function in aFTLD-U patients [47].

The second dysregulated pathway, significantly upregulated in aFTLD-U patients, centered around DNA and RNA transcription and translation. Given the multifunctional role of FET proteins as DNA/RNA-binding proteins involved in various cellular processes such as transcription regulation, RNA splicing and transport, DNA repair, and damage response, alterations in these pathways were not unexpected and likely also relate to the loss of functional (nuclear) FUS, TAF15 and other aggregating proteins in disease.

The final dysregulated pathway involved the upregulation of *GLI1* and *PTCH1,* key components of the Shh signaling pathway. The Shh pathway is critical for cell growth and differentiation and has emerged as a modulator in adult neural tissues through mechanisms such as neurogenesis, anti-oxidation, anti-inflammation, and autophagy [13]. Some studies have additionally suggested that the Shh pathway regulates key functional properties of astrocytes and their modulation of neuronal activity [23, 36], which aligns with our findings in aFTLD-U, where many of the genes with altered expression showed astrocyte-enriched expression. Moreover, it has been shown that dysregulation of the Shh signaling pathway can contribute to mitochondrial dysfunction and neuronal apoptosis [83, 104]. Furthermore, previous studies have shown that the activation of the Shh pathway reduces mitochondrial damage and protects neurons against oxidative stress and apoptosis in autism, PD, stroke, and Down syndrome [1, 17, 25, 39–41, 94, 110], further linking the dysregulated pathways we identified in aFTLD-U.

Importantly, proteins of the GLI family are translocated to the nucleus by TNPO1 upon Shh activation [31, 45, 95], resulting in the transcriptional activation of their target genes. These transcription factors also autoregulate PTCH1 and GLI1 [8]. Moreover, TNPO1 itself is transcriptionally activated by the active form of GLI proteins [67, 79], and GLI1 and FUS were suggested to be transcriptional targets of each other [6, 117]. Thus, GLI, FET proteins, and TNPO1 are interconnected, and defects in any of the steps in the Shh-PTCH-GLI pathway could potentially lead to the accumulation of TNPO1 and the proteins that depend on TNPO1 for nuclear import, including the FET proteins, which are found to accumulate in aFTLD-U. A suggested disease mechanism would be that GLI proteins are retained in the cytoplasm in aFTLD-U patients where TNPO1 is dysfunctional, leading to increased transcription of PTCH1 and an expected further increase in GLI1 expression to compensate for the insufficient nuclear GLI1 protein levels, placing TNPO1 dysfunction at a key regulatory position in FTLD-FET pathogenesis. Dysregulation of the Shh pathway may also contribute to the astrocytic changes we observed, increasing neuronal vulnerability and degeneration in aFTLD-U. Due to the feedback loops in this pathway, it is difficult to distinguish cause from consequence, and further experiments are necessary to test this hypothesis.

In addition to altered expression we also observed differential splicing in aFTLD-U patients versus controls, in line with the important role of FET proteins in RNA processing and splicing [57, 91]. While no significant pathways were identified based on the 227 unique differentially spliced genes, splicing changes were observed in multiple genes involved in myelination and cytoskeletal organization, including *CLDND1*, *MBP*, and *FYN*. Moreover, a near significant increase was identified for oligodendrocyte-enriched genes among the differentially spliced genes. Specifically, from the 23 differentially spliced genes that were found to be enriched in a specific cell-type, 15 were enriched in oligodendrocytes. This unexpected finding highlights the potential vulnerability of oligodendrocytes to FET protein dysfunction and suggests a possible role for these cells in the pathogenesis of FTLD-FET. This is particularly interesting, as in all types of FTLD-FET, including aFTLD-U, there are glial cytoplasmic inclusions in cerebral white matter in cells with the morphology of oligodendrocytes [66, 75], further highlighting the significance of these findings. *CLDND1*, highly expressed in oligodendrocytes [118], was the most significantly differentially spliced gene in our analysis. Although its precise function in the brain remains poorly understood, CLDND1 is known to contribute to the structural integrity of the blood–brain barrier [68, 96]. The altered splicing of *CLDND1* may reflect changes in oligodendrocyte-endothelial interactions or blood–brain barrier homeostasis, a phenomenon that has been implicated in the pathophysiology of neurodegenerative disorders. We also observed significant alternative splicing in Myelin basic protein (*MBP*), encoding a key structural protein of the myelin sheath, synthesized by oligodendrocytes in the central nervous system [30]. Alternative splicing of the *MBP* transcript results in different isoforms of myelin proteins, which are differentially expressed during development and myelination [9, 33, 89]. MBP also regulates the glial cytoskeleton and functions similarly to microtubule-associated proteins such as tau [35]. This suggests that MBP dysregulation may contribute to cytoskeletal instability, which is a hallmark of neurodegenerative diseases. Supporting this, the PPI network revealed a cluster of proteins involved in cytoskeletal structure, with FYN serving as a central node. Additionally, MBP has been shown to bind to FYN [82, 101]. FYN is involved in several signaling pathways during oligodendrocyte development and myelination [55, 70, 103]. In particular, FYN has been postulated to be a key regulatory element in the myelination process, triggering the phosphorylation of hnRNPs responsible for the efficient transport of *MBP* mRNA to the site of developing oligodendrocyte processes in contact with neurons [38, 55, 70, 93, 115]. Given that hnRNP dysfunction is implicated in aFTLD-U, disruptions in *MBP* mRNA transport may also contribute to disease pathology. While FYN is important for *MBP* mRNA transport, it also regulates additional processes essential for oligodendrocyte growth and myelination through three major downstream pathways, influencing Rho-family GTPase signaling, microtubule cytoskeleton dynamics, and MBP translation [46, 49, 112]. These interconnected processes highlight the central role of FYN in maintaining oligodendrocyte function and myelin integrity. Immunohistochemistry for MBP also showed a significant reduction in myelin staining in the aFTLD-U patients, which was confirmed by LFB/HE staining, possibly reflecting a primary white matter involvement in disease pathogenesis.

Together, these findings reinforce the evolving understanding of glial cell functions. For a long time, glial cells have been considered supportive of neurons in the mammalian brain [34]. However, extensive research has revealed their diverse roles in neural function, development, and disease, and defects in glial cells are already implicated in many neurological diseases [28]. Dysregulation of astrocytes and oligodendrocytes in various pathological conditions may reflect a compensatory response to neuronal damage or broader disruptions in the central nervous system; however, it is also possible that the pathological process may originate within glial cells, highlighting their potential as primary drivers of neurodegeneration. Future functional studies are needed to investigate how alterations in astrocytes and oligodendrocytes may modulate neuron-glial interactions in disease, including in aFTLD-U. Understanding these complex glial interactions is essential for developing targeted therapies for neurodegenerative and neuroinflammatory diseases.

A limitation of our study is that bulk RNA sequencing lacks the resolution to fully capture cell-type specific gene expression or splicing events. Although we adjusted for differences in the cell-type composition of our samples, this approach cannot entirely resolve such effects. Furthermore, aFTLD-U patients and control cohorts were not matched for age and sex. While we included these variables as covariates in our differential gene expression and differential splicing studies, we cannot exclude that these differences may have impacted our results. Moreover, short-read RNA sequencing has limitations in accurately resolving complex alternative splicing events. Long-read sequencing technologies can help in identifying and interpreting alternative splicing events. Therefore, future studies should investigate the expression levels of the identified pathways in specific cell types to elucidate which cell populations drive the detected associations (e.g., using purified cell populations or single-nuclei sequencing), preferably with long-read sequencing.

To conclude, transcriptional analyses confirmed alterations in genes involved in DNA binding and transcriptional regulation, as well as in mitochondrial pathways, highlighting the crucial roles of FET proteins in these biological processes. We further report a novel link between aFTLD-U and alterations in key regulators of the Shh signaling pathway, as well as the involvement of astrocytes and oligodendrocytes in the disease process. Additionally, we observed a significant reduction in myelin levels in aFTLD-U patients compared to those in the control group. Overall, this study highlighted transcriptional changes in non-neuronal cell types in patients with aFTLD-U.

## Supplementary Information

Below is the link to the electronic supplementary material.

Supplementary file1 (DOCX 12236 KB)

Supplementary file2 (XLSX 2079 KB)

## Data Availability

The metadata, gene expression counts, and splicing data (per individual counts) are available in the Synapse platform under accession number “syn53181336”. All other relevant data supporting the key findings of this study are available in the article and the Supplementary materials.

## Abbreviations

FTLD: Frontotemporal lobar degeneration
bvFTD: Behavioral-variant frontotemporal dementia
svPPA: Semantic variant primary progressive aphasia
nfvPPA: Nonfluent variant primary progressive aphasia
PSP: Progressive supranuclear palsy
CBS: Corticobasal syndrome
TDP-43: TAR DNA-binding protein 43
FUS: Fused in sarcoma
aFTLD-U: Atypical FTLD with ubiquitin inclusions
NIFID: Neuronal intermediate filament inclusion body disease
BIBD: Basophilic inclusion body disease
hnRNP: Heterogeneous nuclear ribonucleoprotein
TNPO1: Transportin 1
EWS: Ewing’s sarcoma protein
TAF15: TATA-binding protein-associated factor 15
Cryo-EM: Cryogenic electron microscopy
Shh: Sonic hedgehog
RPKM: Reads per kilobase per million mapped reads
RIN: RNA integrity number
PPI: Protein–protein interaction
DEG: Differentially expressed genes
WGCNA: Weighted gene co-expression network analysis
nTPM: Normalized expression values
OPCs: Oligodendrocyte precursor cells
qPCR: Quantitative PCR
ΔPSI: Delta percent spliced in
LFB/HE: Luxol fast blue stain with hematoxylin and eosin counterstain
