# Supplementary material for: Brain transcriptomics highlight abundant gene expression and splicing alterations in non-neuronal cells in aFTLD-U
Source: Acta Neuropathol. 2025 Aug 10;150(1):17. doi: 10.1007/s00401-025-02919-x (PMC12336083; doi:10.1007/s00401-025-02919-x)
Supplement: Supplementary file 1 — Supplementary file1 (DOCX 12236 KB) [file 401_2025_2919_MOESM1_ESM.docx]

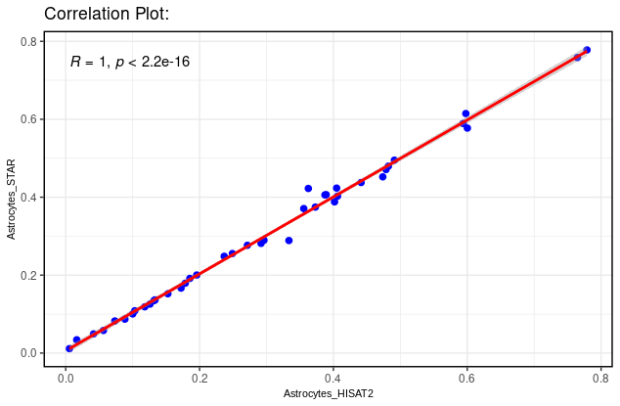

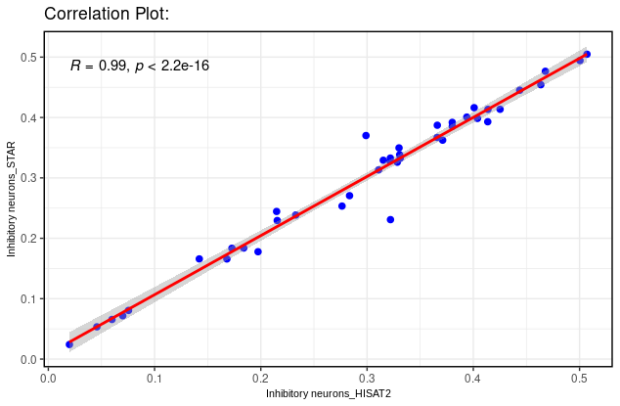

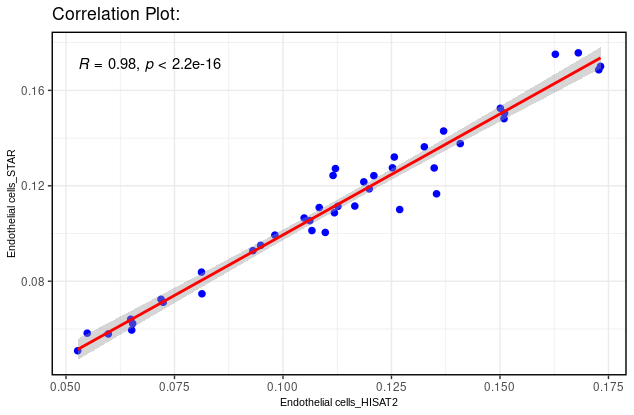


b

a


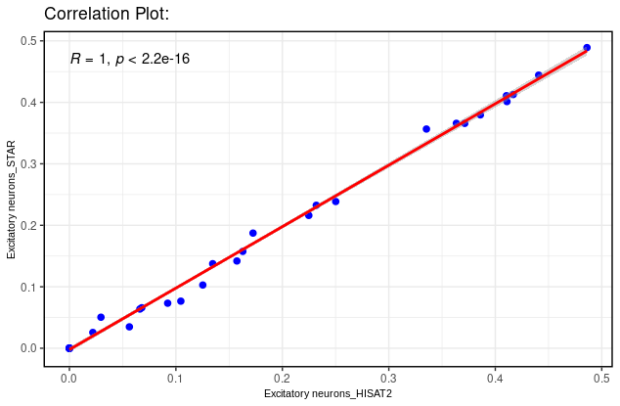


c

d


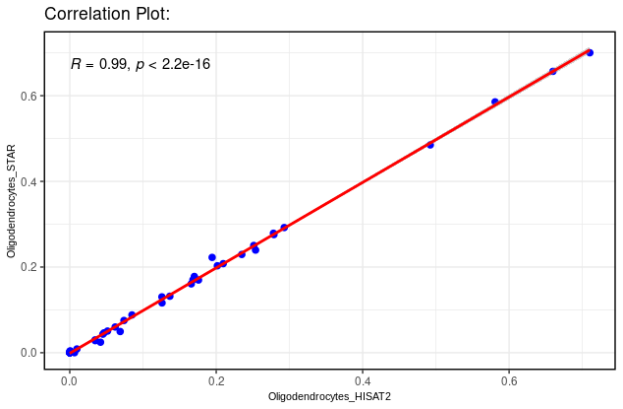


e

**Supplementary Figure 1. Comparison of cell-type proportion estimates from HISAT2- and STAR-aligned reads.** Cell-type proportions were estimated using CIBERSORTx on RNA-seq data aligned with either HISAT2 or STAR. Scatter plots show the comparison between HISAT2- and STAR-derived estimates for each major brain cell type: (a) Astrocytes (Pearson R=1, *P-value*<2.2e-16), (b) Endothelial cells (Pearson R=0.98, *P-value*<2.2e-15), (c) Inhibitory neurons (Pearson R=0.99, *P-value*<2.2e-16), (d) Excitatory neurons (Spearman R=1, *P-value*<2.2e-16), and (e) Oligodendrocytes (Spearman R=0.99, *P-value*<2.2e-16). Each blue dot represents a sample, and the red line indicates the linear regression fit with a 95% confidence interval shaded in gray. Correlation coefficients (R) and associated *P-values* are shown in each panel, demonstrating strong concordance across all cell types.


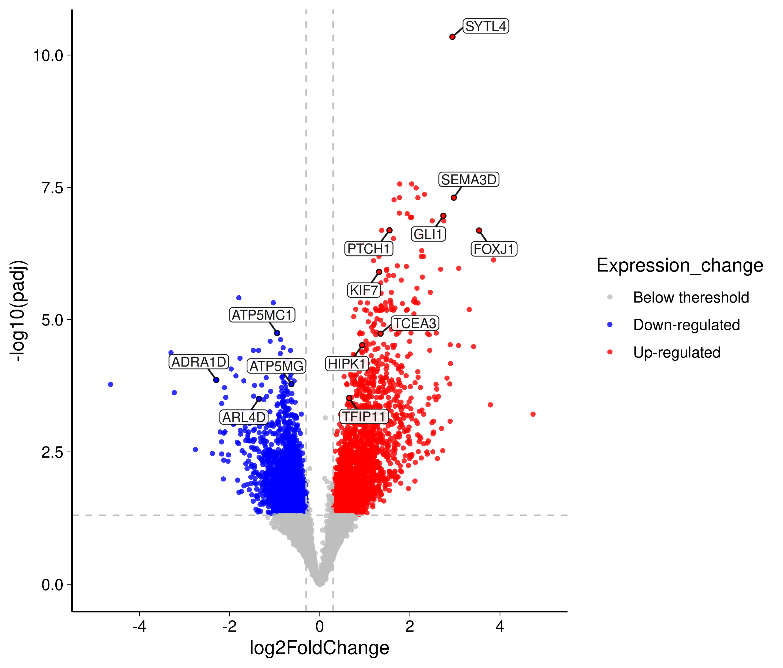


**Supplementary Figure 2. Differential gene expression in aFTLD-U patients versus control individuals without cell-type adjustment.** Volcano plot represents the differentially expressed genes in aFTLD-U patients versus control individuals, without adjustment for cell-type proportions. The fold change is presented in a log2 scale on the x-axis, while the adjusted *P-value* is presented on the y-axis on a −log10 scale.


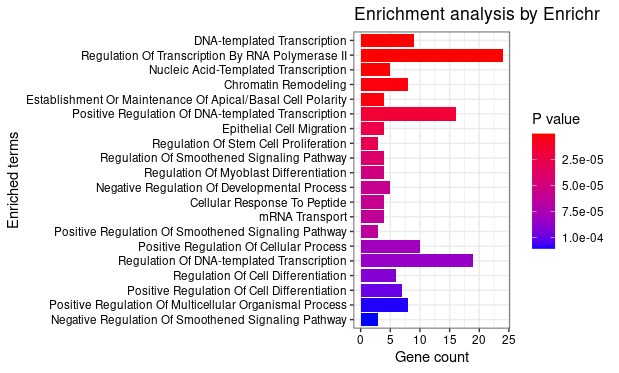

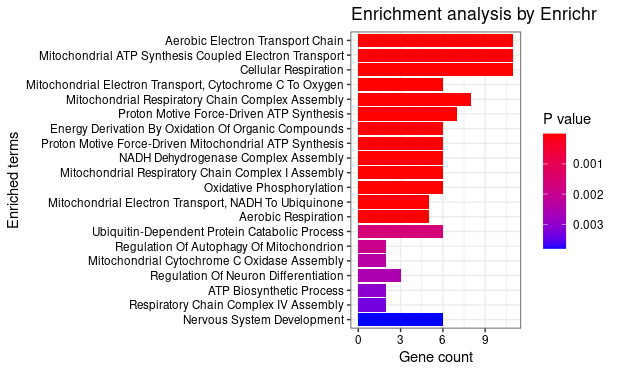


b

a


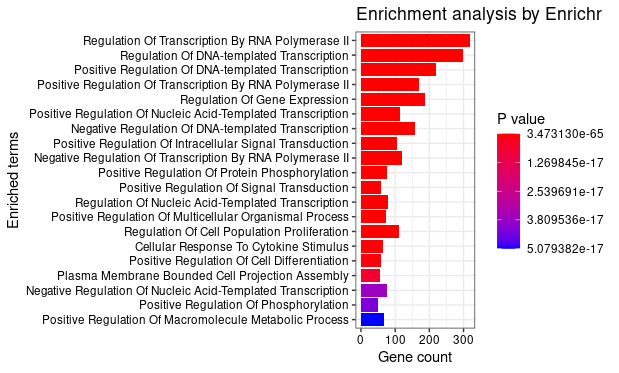

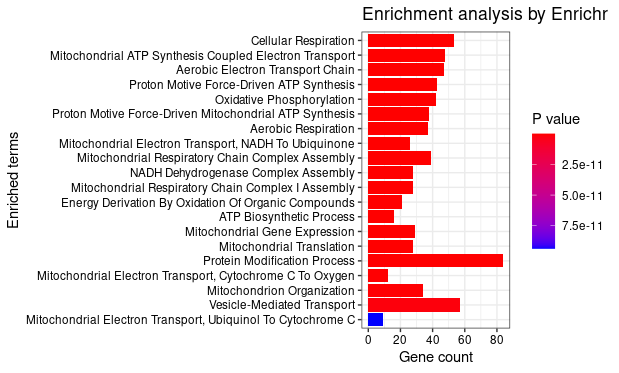


d

c

**Supplementary Figure 3. Pathway enrichment analysis of differentially expressed genes in aFTLD-U patients versus control individuals. (a)** Top 20 GO biological process terms for up-regulated genes in comparing aFTLD-U patients to control individuals, with adjusting for cell type proportions. **(b)** Top 20 GO biological process terms for down-regulated genes in comparing aFTLD-U patients to control individuals, with adjusting for cell type proportions. **(c)** Top 20 GO biological process terms for up-regulated genes in comparing aFTLD-U patients to control individuals without adjusting for cell type proportions. **(d)** Top 20 GO biological process terms for down-regulated genes in comparing aFTLD-U patients to control individuals without adjusting for cell type proportions.

**
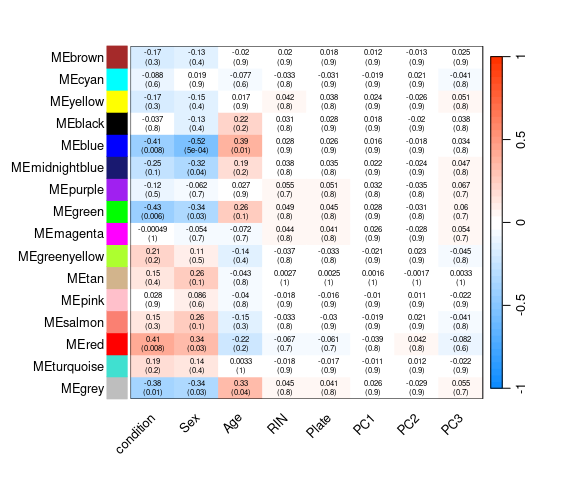
**

a

c

b

**
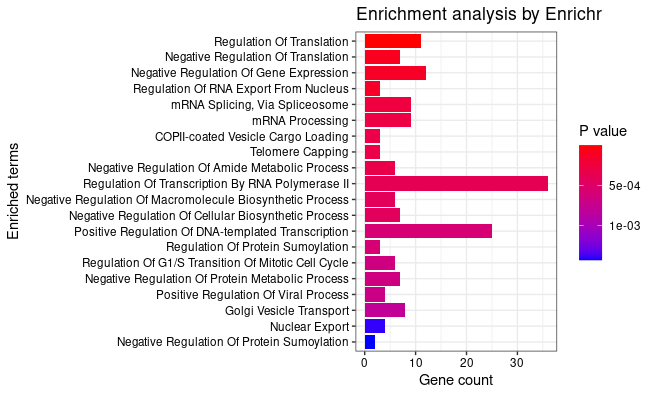

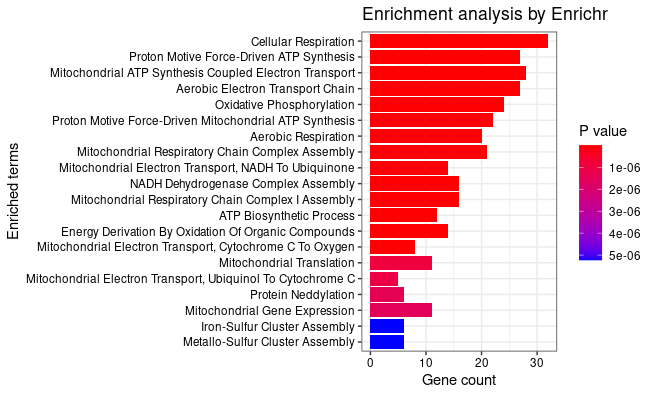
**

**Supplementary Figure 4. WGCNA Co-expression analyses of aFTLD-U patients versus control individuals using residual expression values.** For these analyses the residual expression values used as input for the WGCNA were adjusted for RIN, Plate (= experimental batch), and the first three principal components of the proportions of cell types, not for sex and age at death. **(a)** Module-trait relationships are presented for aFTLD-U patients and control individuals. Modules with co-expressed genes, either upregulated (red) or downregulated (blue) in relation to each traits, are shown with a unique name (color) attributed to each of the modules. Correlations and *P-values* are shown for each variable of interest, including condition (aFTLD-U patients and control individuals), sex, age at death, RNA integrity number (RIN), plate (=experimental batch), and principal components of cell type proportions. **(b)** Pathway enrichment of the green module. The top 20 terms from the GO analysis of biological processes are shown. **(c)** Pathway enrichment of the blue module. The top 20 terms from the GO analysis of biological processes are shown. **(d)** Pathway enrichment of the red module. The top 20 terms from the GO analysis of biological processes are shown.


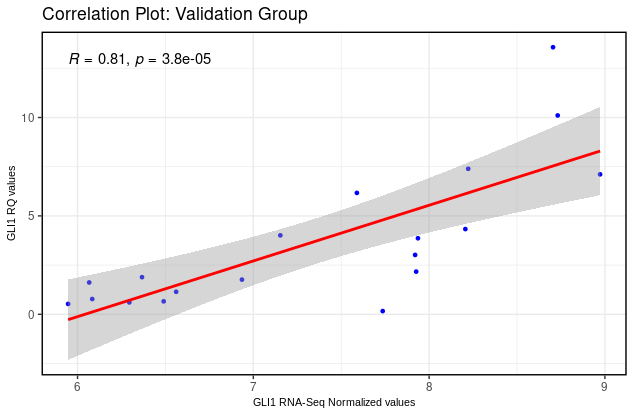


**Supplementary Figure 5. Correlation between normalized values obtained from qPCR and bulk RNA sequencing for *GLI1*.** The correlation plot showed a significant positive correlation between *GLI1* normalized values from RNA-seq and *GLI1* RQ values from qPCR (Spearman R=0.81, *P-value*=3.8e-05). Each blue dot represents an individual sample, and the red regression line with a shaded confidence interval.


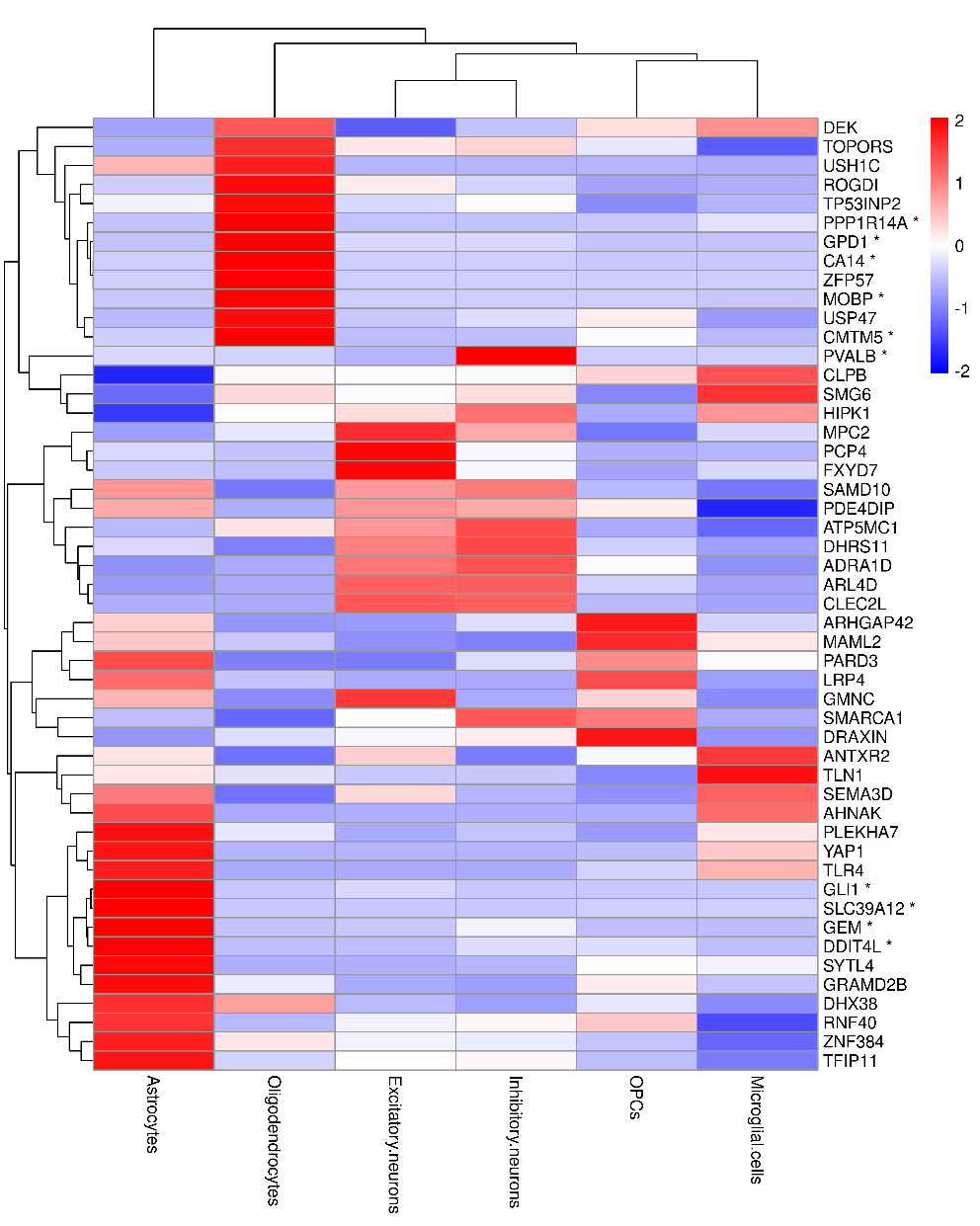


**Supplementary Figure 6. Cell type-enriched expression of the top 50 differentially expressed genes.** The heatmap displays the row-scaled expression values of the top 50 differentially expressed genes across 6 major brain cell types, based on nTPM values extracted from the Human Protein Atlas. Genes significantly enriched in specific cell types are indicated with an asterisk (*). Notably, many genes show high expression in astrocytes and oligodendrocytes, including 4 astrocyte-enriched and 5 oligodendrocyte-enriched genes.


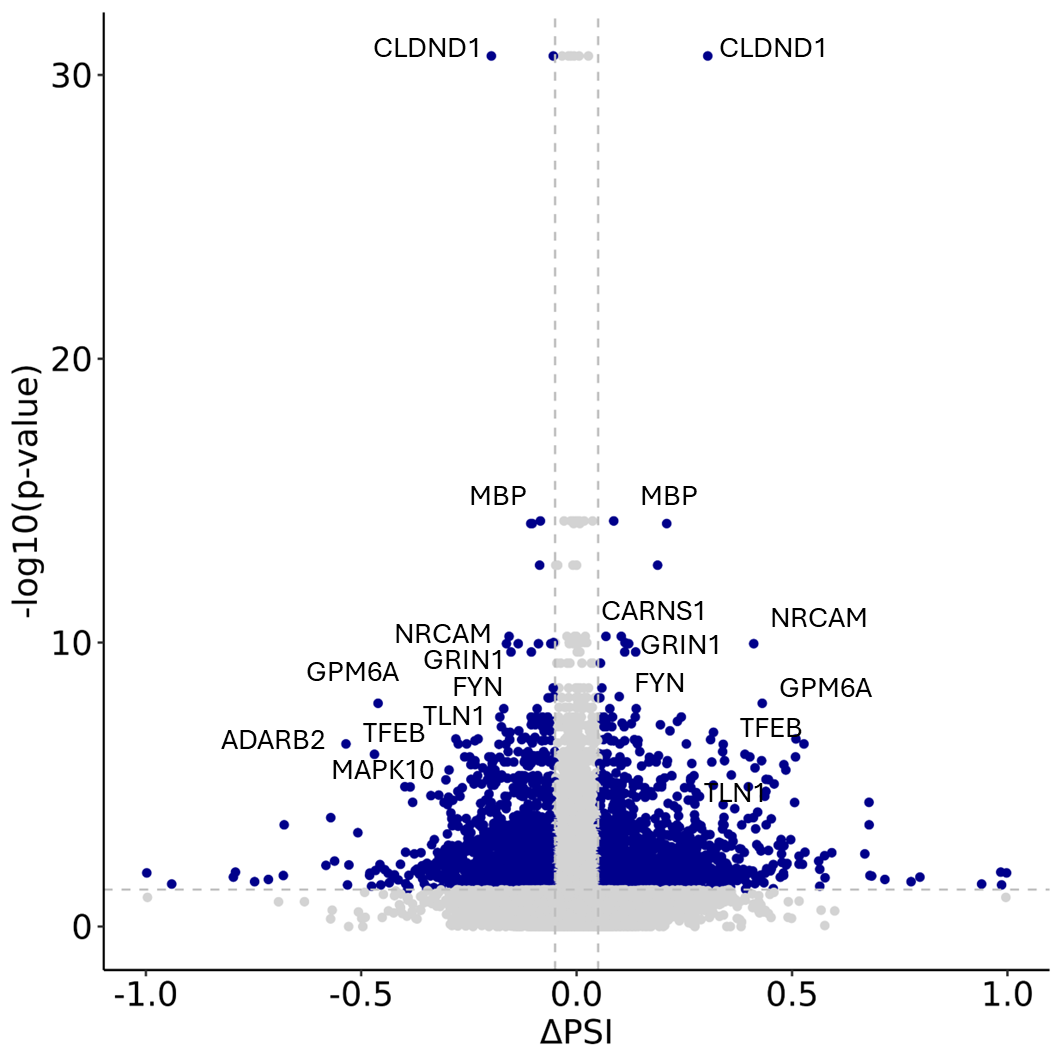


**Supplementary Figure 7. Differential splicing in aFTLD-U patients versus control individuals without cell-type adjustment.** The volcano plot represents the differentially spliced events. In dark blue, events within a significant cluster (FDR<0.05) and a |ΔPSI|≥5%.

**
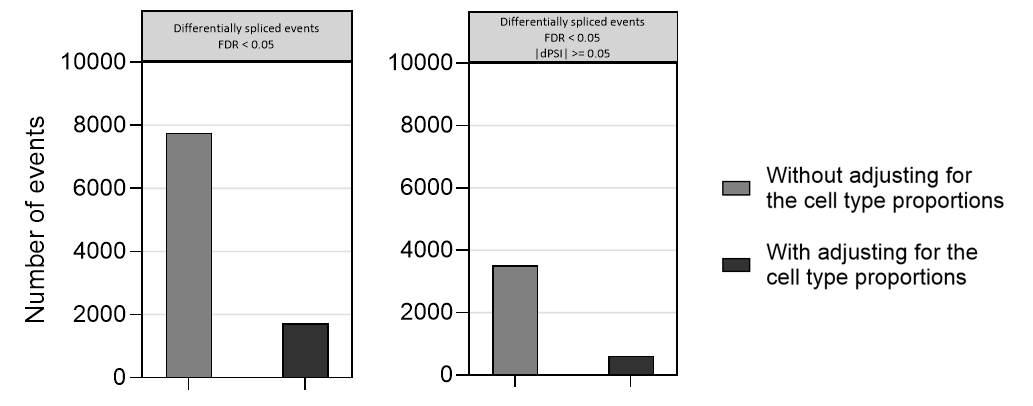
**

a

b

c

**
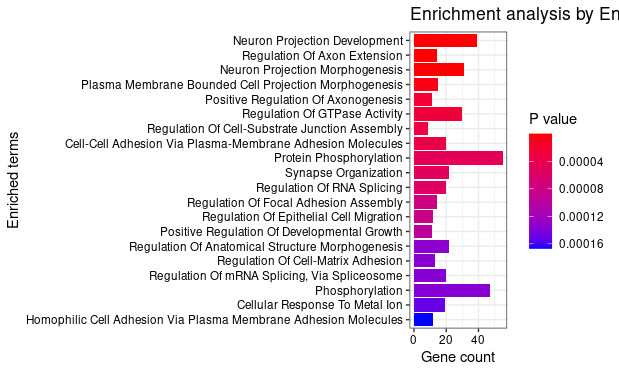

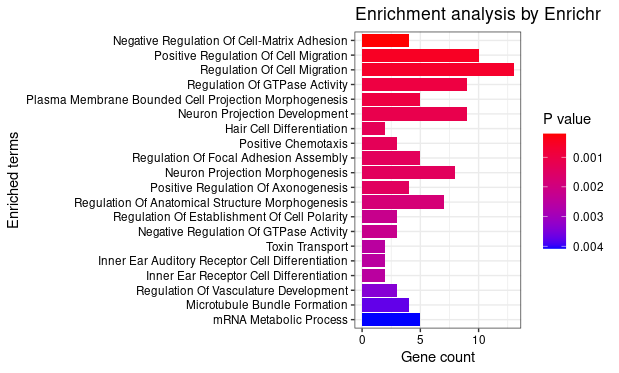
**

d

**Supplementary Figure 8. Impact of cell-type proportion adjustment on splicing event detection and pathway enrichment. (a)** Bar plots showing the number of differentially spliced events detected before and after adjusting for cell-type proportions. The left panel represents events identified at FDR<0.05, while the right panel shows events after setting the threshold for ΔPSI (|ΔPSI|≥5% & FDR<0.05). Adjusting cell-type proportions substantially reduces the number of detected events. **(b)** Proportion of different splicing events identified before and after adjusting for cell-type proportions. The majority of events are annotated splicing events (dark blue), with smaller fractions representing novel splicing events, including novel annotated pairs (light blue), cryptic 5’ (red), cryptic 3’ (green), cryptic unanchored events (yellow), and unknown strand assignments (gray). **(c)** Top 20 GO biological process terms for significant spliced genes without adjusting for cell type proportions. **(d)** Top 20 GO biological process terms for significant spliced genes with adjusting for cell type proportions.


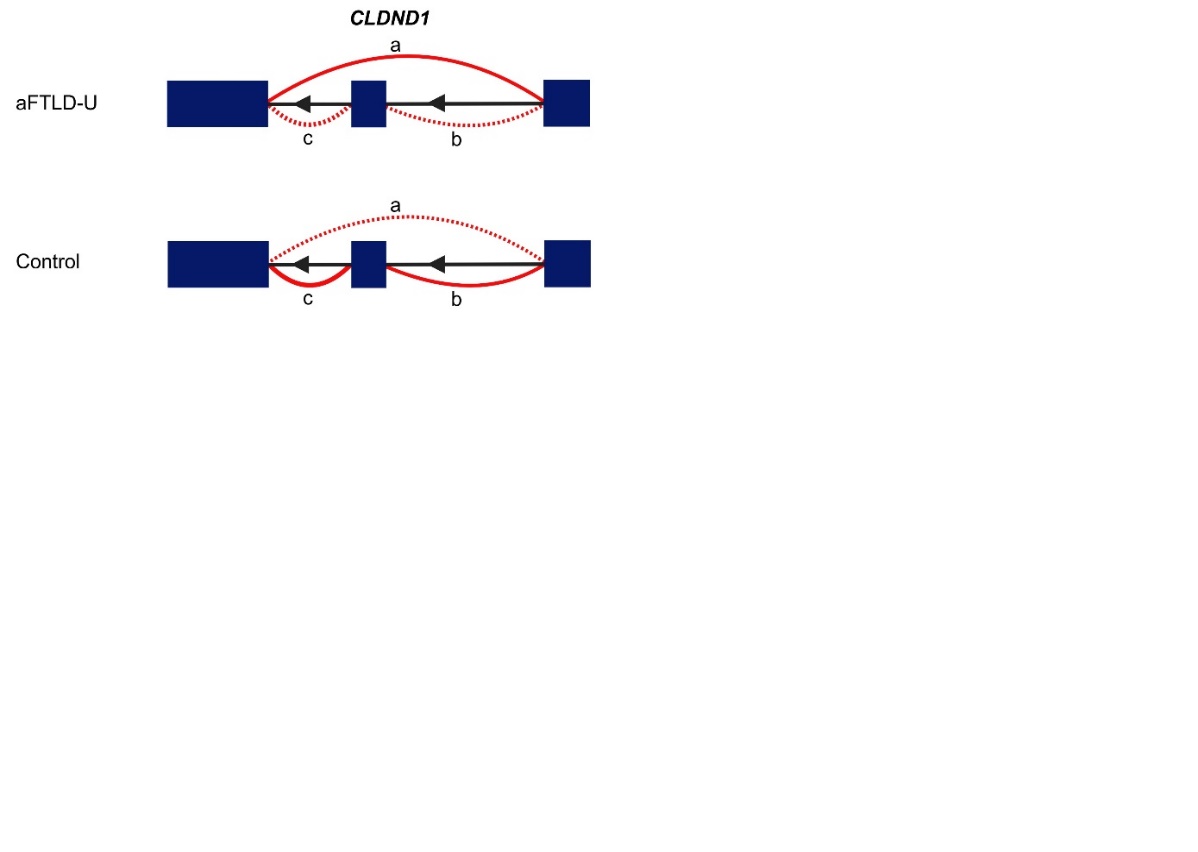


aFTLD-U

Control

***CLDND1***

a


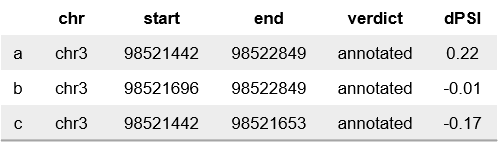


b

d

c


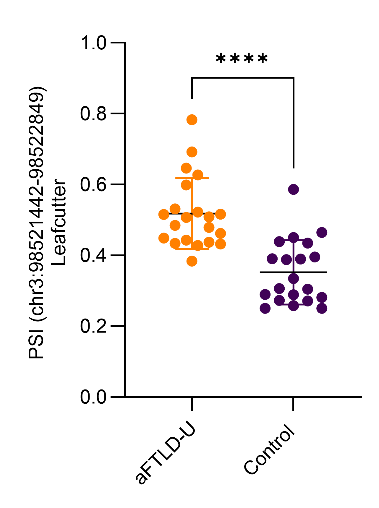

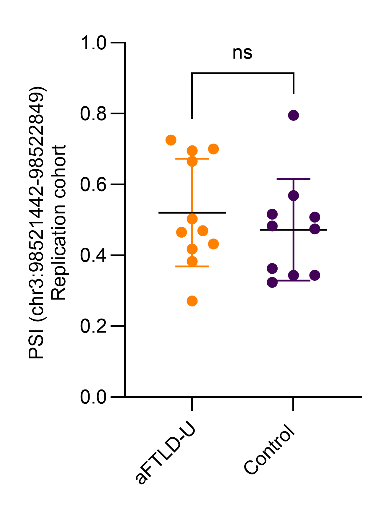

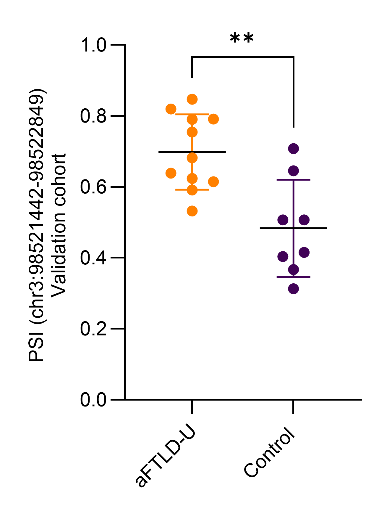


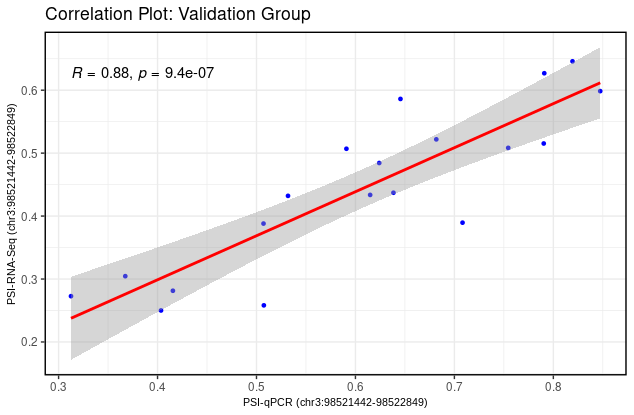


e

**Supplementary Figure 9. *CLDND1* Splicing Alterations. (a)** Schematic representation of the splicing events observed in *CLDND1* between aFTLD-U cases and controls. Exons are represented as dark blue boxes, and splice junctions are shown as curved lines. Red lines indicate splice junctions with increased usage, whereas dotted red lines indicate junctions with decreased usage. Below each schematic, tables display the chromosomal coordinates of the splice junctions, their classification, and the corresponding ΔPSI values. **(b)** PSI (Percent Spliced-In) values for the *CLDND1* splicing event (chr3:98521442-98522849) obtained from LeafCutter analysis showed a significant increase in aFTLD-U cases compared to controls (Data represented as mean±SD, Mann-Whitney test, *P-value*<0.0001). **(c)** qPCR validation of the *CLDND1* splicing event (chr3:98521442-98522849), confirming increased inclusion in aFTLD-U cases (Data represented as mean±SD, t-test, *P-value*=0.0012). **(d)** Replication of qPCR results for *CLDND1* splicing events. No significant difference was observed between aFTLD-U cases and controls in the replication cohort (Data represented as mean±SD, t-test, *P-value*=0.45). **(e)** Correlation between PSI values obtained from qPCR and bulk RNA sequencing (LeafCutter) for *CLDND1***.** Each blue dot represents an individual sample, and the red regression line with a shaded confidence interval demonstrates a strong correlation (*CLDND1*: Pearson correlation, R=0.88, *P-value*=9.4e-07). **(f)** Combined analysis of the validation and replication cohorts for *CLDND1*. When data from both cohorts were combined, significant differences in splicing were observed between aFTLD-U cases and controls (*P < 0.01*).

a

aFTLD-U


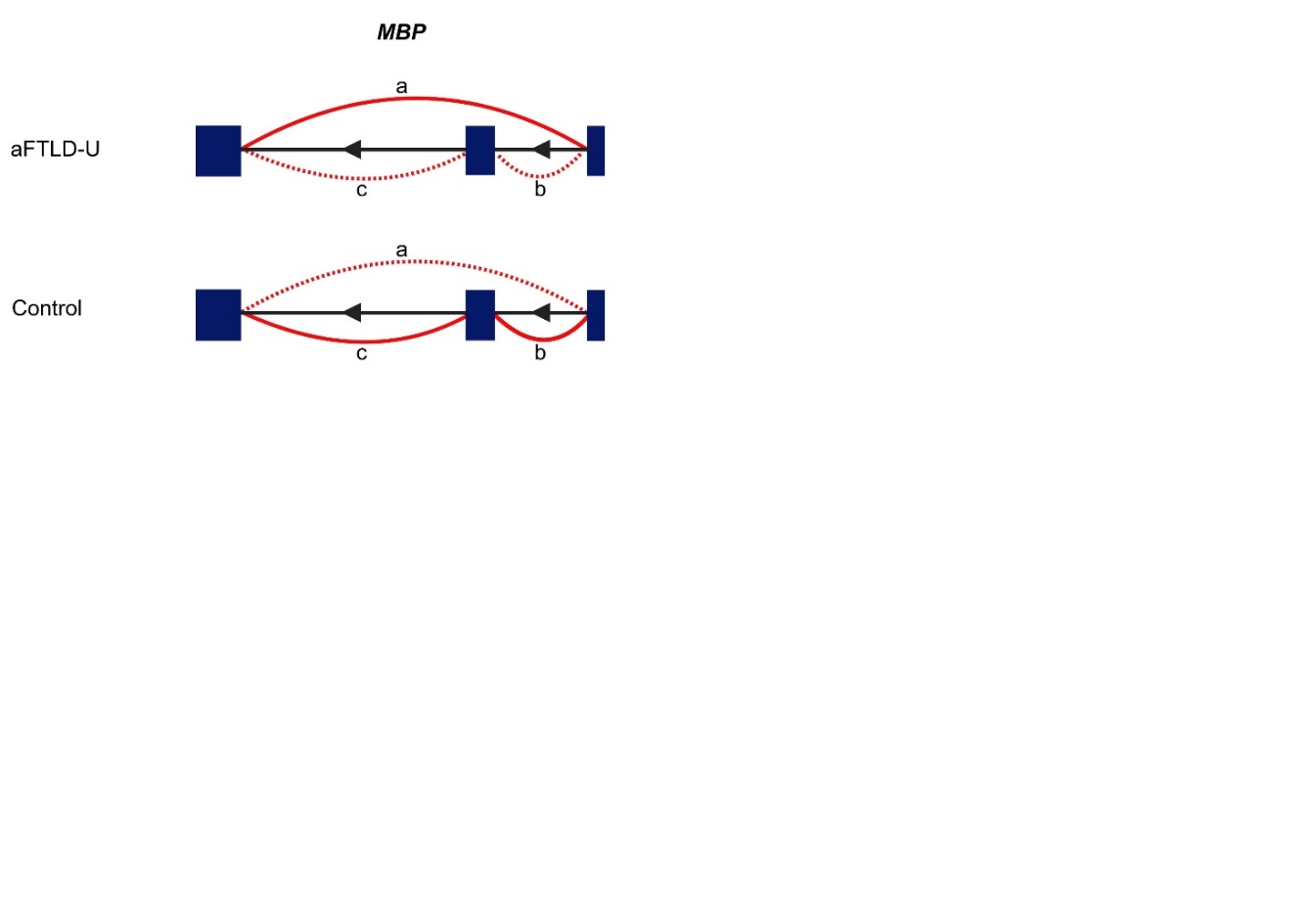


***MBP***

Control

**
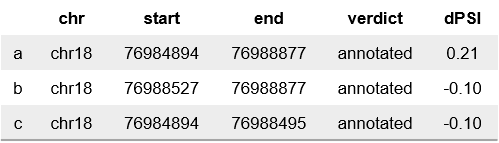
**


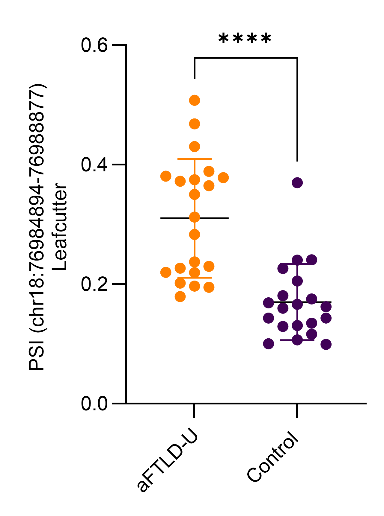

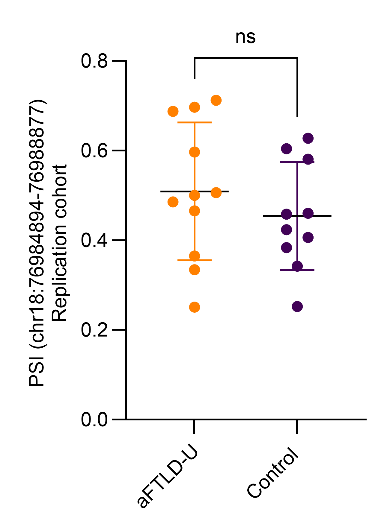

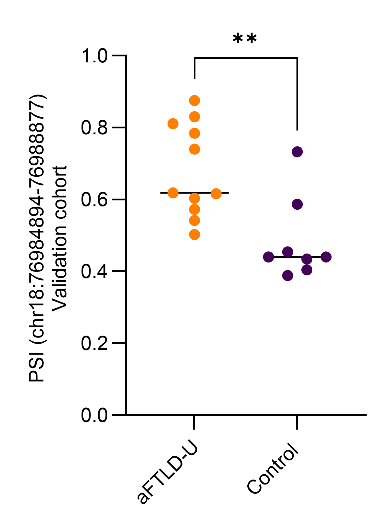


d

c

b

e


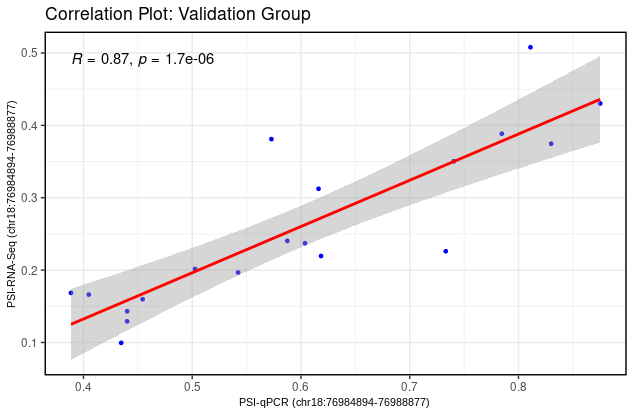


**Supplementary Figure 10. *MBP* Splicing Alterations. (a)** Schematic representation of the splicing events observed in *MBP* between aFTLD-U cases and controls. Exons are represented as dark blue boxes, and splice junctions are shown as curved lines. Red lines indicate splice junctions with increased usage, whereas dotted red lines indicate junctions with decreased usage. Below each schematic, tables display the chromosomal coordinates of the splice junctions, their classification, and the corresponding ΔPSI values. **(b)** PSI (Percent Spliced-In) values for the *MBP* splicing event (chr18:76984894-76988877) obtained from LeafCutter analysis showed a significant increase in aFTLD-U cases compared to controls (Data represented as mean±SD, Mann-Whitney test, *P-value*<0.0001). **(c)** qPCR validation of the *MBP* splicing event (chr18:76984894-76988877), confirming increased inclusion in aFTLD-U cases (Data represented as mean±SD, Mann-Whitney test, *P-value*=0.0025). **(d)** Replication of qPCR results for *MBP* splicing events. No significant difference was observed between aFTLD-U cases and controls in the replication cohort (Data represented as mean±SD, t-test, *P-value*=0.37). **(e)** Correlation between PSI values obtained from qPCR and bulk RNA sequencing (LeafCutter) for *MBP*. Each blue dot represents an individual sample, and the red regression line with a shaded confidence interval demonstrates a strong correlation (*MBP*: Pearson correlation, R=0.87, *P-value*=1.7e-06).


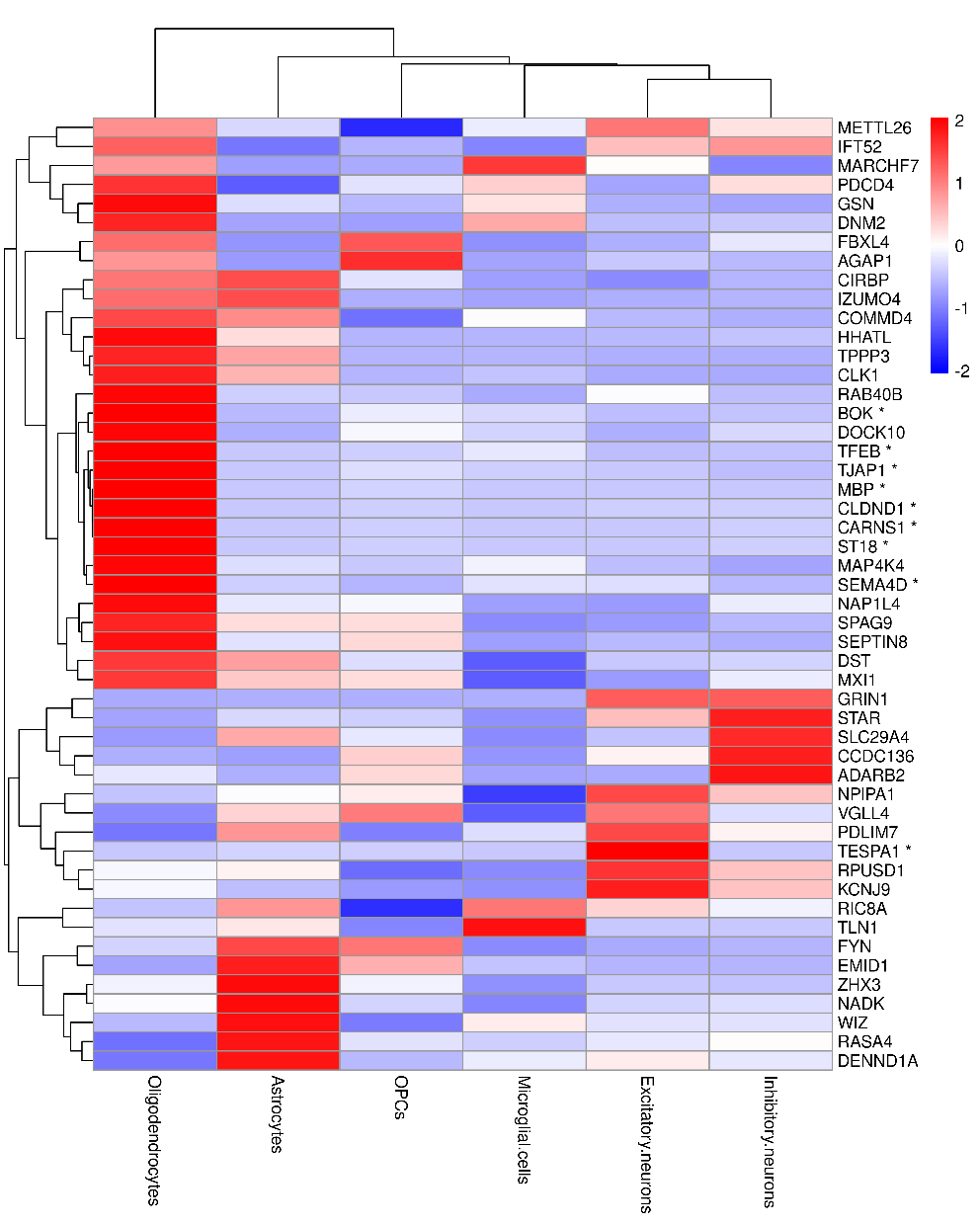


**Supplementary Figure 11. Cell type-enriched expression of the top 50 differentially spliced genes.** The heatmap displays the row-scaled expression values of the top 50 differentially spliced genes across 6 major brain cell types, based on nTPM values extracted from the Human Protein Atlas. Genes significantly enriched in specific cell types are indicated with an asterisk (*). 24 of the 50 genes show predominant expression in oligodendrocytes, including 4 genes that are specifically enriched in this cell type.


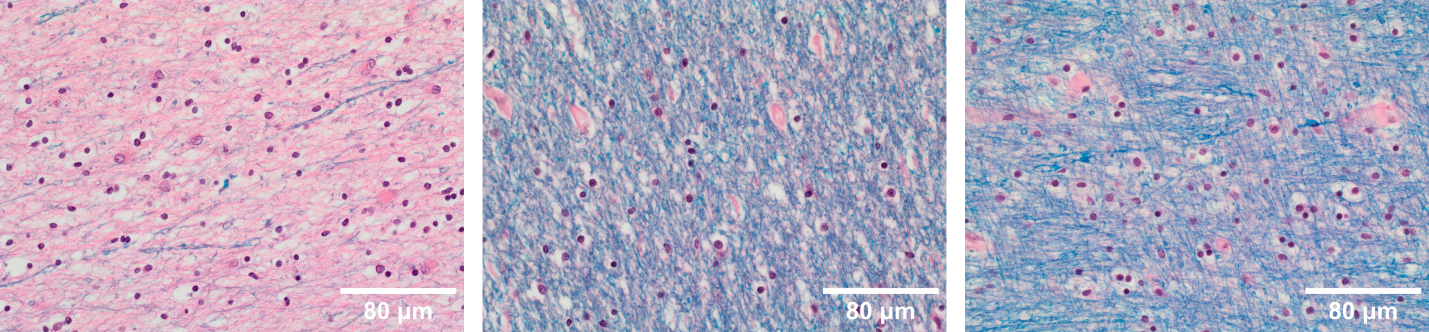


b

c

a

**Supplementary Figure 12. Myelin integrity in control and disease cases visualized by Luxol Fast Blue/Hematoxylin and Eosin (LFB/HE) staining.** Representative images of formalin-fixed, paraffin-embedded brain tissue sections (6 μm thick) stained with Luxol Fast Blue with hematoxylin and eosin counterstain (LFB/HE) to assess myelin distribution. **(a)** aFTLD-U case showing marked reduction in myelin staining, indicating severe demyelination **(b)** control brain showing dense myelin staining (blue) **(c)** FTLD-TDP case showing some mild loss of myelin integrity. Myelin is stained blue, nuclei are purple, and background axons and glial elements are pink. All images are acquired at 40× magnification. Scale bar = 80 μm.
